# ﻿Morphological and phylogenetic analyses reveal novel entomopathogenic fungi infecting scale insects and aphids in China

**DOI:** 10.3897/imafungus.16.170123

**Published:** 2025-10-08

**Authors:** Chunlin Yang, Xiulan Xu, Xinyue Li, Feng Liu, Zhen Zeng, Qiangang Xiao, Yinggao Liu

**Affiliations:** 1 College of Forestry, Sichuan Agricultural University, Chengdu 611130, China; 2 National Forestry and Grassland Administration Key Laboratory of Forest Resources Conservation and Ecological Safety on the Upper Reaches of the Yangtze River, College of Forestry, Sichuan Agricultural University, Chengdu 611130, China; 3 Forestry Research Institute, Chengdu Academy of Agricultural and Forestry Sciences, Chengdu 611130, China

**Keywords:** 6 new taxa, *
Cladosporiaceae
*, *
Clavicipitaceae
*, fungal entomopathogens, *
Podonectriaceae
*

## Abstract

Entomopathogenic fungi exhibit a cosmopolitan distribution across diverse ecosystems, with their ubiquitous presence intrinsically linked to insect habitats—essentially occurring wherever insect populations exist. These fungi represent a vital biological resource, particularly in agriculture and forestry. They serve as a crucial repository of fungal strains for biological pest control. This investigation identified seven species from southwest China based on multi-gene (ITS, LSU, SSU, *act*, *rpb*1, *rpb*2, and *tef*1-α) phylogenetic analyses and morphological characteristics, including one new species and one newly recorded species in *Cladosporium (Cladosporiaceae)*; four new species in *Moelleriella (Clavicipitaceae)*; and one new species in *Podonectria (Podonectriaceae)*. All seven fungi are in their asexual morphs and were discovered on aphids or scale insects, which are common and significant pests. These include *Cladosporium kuwanaspidis*, *Cladosporium guizhouense*, *Moelleriella eucalypti*, *Moelleriella boehmeriae*, *Moelleriella cinnamomum*, *Moelleriella citrus*, and *Podonectria multiarmata*. Descriptions and illustrations for all seven taxa are provided. Six of these species were collected from scale insects, specifically those found in bamboo forests, broad-leaved forests, or understory vegetation, and one was collected from aphids, primarily distributed on the underside of night-blooming jasmine leaves. This work reveals the rich diversity of entomopathogenic fungi in southwestern China, not only on larger insects such as Lepidoptera and Hymenoptera but also on smaller Hemiptera, many of which are significant agricultural and forestry pests. This study contributes fungal resources that may support the development of innovative pest control methods in the future.

## ﻿Introduction

To ensure environmental safety, fungal agents are considered key biological regulators in the application of insecticides for pest control (Roy and Cottrell 2008; Sharma and Sharma 2021). The initial recommendation to use microbial insect pathogens for controlling insects was proposed by LeConte (1874) and Pasteur (1874). Subsequently, the first successful application of *Metarhizium anisopliae* was conducted in Russia against *Bothynoderes punctiventris* (*Coleoptera*: *Curculionidae*) by Krassilstschik (1888). This achievement built upon the earlier identification of the microbial agent, initially named *Entomophthora anisopliae*, which targeted *Anisoplia austriaca* (*Coleoptera*: *Scarabaeidae*).

To date, more than 1,000 species of entomopathogenic fungi have been documented across over 100 genera (Hywel-Jones 1993; Vega et al. 2012; Hawksworth and Lücking 2017; Corallo et al. 2019; Wu et al. 2024). Among these species, such as *Akanthomyces* Lebert, *Beauveria* Vuill., *Cordyceps* Fr. (=*Isaria* Pers.), *Lecanicillium* W. Gams & Zare, and *Metarhizium* Sorokin, are widely recognized for their potential in biological control. Notably, species like *A. aculeatus*, *B. bassiana*, *B. brongniartii*, *C. fumosorosea*, *L. lecanii* (≡*Verticillium lecanii*), *L. muscarium*, *L. longisporum*, and *M. anisopliae* are commonly employed in pest management strategies (Faria and Wraight 2007; McKinnon et al. 2017; Corallo et al. 2019; Stone and Bidochka 2020; Nicoletti and Becchimanzi 2020; Nishi et al. 2021; Khonsanit et al. 2024). For example, 171 products have been developed as biocontrol agents, primarily based on *B. bassiana*, *B. brongniartii*, *C. fumosorosea*, and *M. anisopliae* (Faria and Wraight 2007). *Beauveria bassiana*, marketed as “Mycotrol”, has been utilized as a commercial mycoinsecticide to target a broad spectrum of insect pests in North America (Wraight et al. 2021). Similarly, the commercial product “Green Muscle,” derived from *M. acridum* (≡M. anisopliae var. acridum), has proven effective against locust and grasshopper pests in Africa (Niassy and Diarra 2011). In addition, entomopathogenic fungi exhibit diverse nutritional behaviors—functioning as biotrophs, necrotrophs, or hemibiotrophs (Vega et al. 2009)—and demonstrate additional ecological roles, including rhizosphere colonization, fungal endophytism, plant disease antagonism, and enhancement of plant growth or stress resistance (Kabaluk and Ericsson 2007; Kim et al. 2008; Ownley et al. 2008; Vega 2008; Pava-Ripoll et al. 2011; Akutse et al. 2013; Jaber and Enkerli 2017; Jaber and Ownley 2017; Mantzoukas and Eliopoulos 2020; Sharma and Sharma 2021; Muola et al. 2023).

Insect-associated fungi have been widely reported across the extensive regions south of the Yellow River basin, with most species displaying distribution patterns characteristic of tropical, subtropical, and temperate zones (Liang et al. 2016; Chen et al. 2021; Wei et al. 2022; Xiao et al. 2023; Chuang et al. 2024; Wang et al. 2024, 2025), but have been rarely documented in the Sichuan region. The development of entomogenous fungi is closely linked to vegetation and insect populations, as well as climatic and soil conditions (Hajek and Leger 1994; Quesada-Moraga et al. 2007, 2024; Vega et al. 2009; Medo and Cagáň 2011), although experimental validation in this area remains limited. Scattered studies have indicated that entomogenous fungi are more commonly found in areas with rich vegetation, relatively high humidity, suitable temperatures, and shaded environments. Chuang et al. (2024) found that in Taiwan, China, entomogenous fungi belonging to the *Cordycipitaceae* are more readily collected in regions with an average temperature of 19–20.5 °C, rainfall ranging from 2,300 to 3,150 mm, and relative humidity between 81% and 82.5%. Wang et al. (2025) collected and described two new genera and 13 new species within the *Clavicipitaceae*, associated with whiteflies and scale insects on various host plants in Yunnan and Hainan, China.

Sichuan is recognized as one of the key biodiversity research hotspots, owing to its diverse ecosystems, complex habitat conditions, and rich plant species. While the region supports a wide variety of insect fauna, entomogenous fungi have not been systematically studied, and their species diversity is likely significantly underestimated. In our ongoing search for entomogenous fungi associated with miniature insects, several new taxa of entomopathogenic fungi (*Nectriaceae*; *Podonectriaceae*) were isolated from scale insects collected in Sichuan Province, primarily from bamboo and walnut hosts (Xu et al. 2021a; Liu et al. 2023). To further explore the diversity of entomogenous fungi, we conducted field collections at multiple sites across Sichuan. Detailed morphological descriptions are provided along with relevant ecological data.

Comprehensive phylogenetic analyses were performed using a seven-locus dataset (ITS, LSU, SSU, *tef*1-α, *rpb*1, *rpb*2, and *act*) to ensure accurate taxonomic position. This study enriches the entomogenous fungi database in Sichuan and offers valuable strains for sustainable pest control in agriculture and forestry.

## ﻿Materials and methods

### ﻿Fungal collection and isolation

Specimens consisting of whole leaves or branches bearing fruiting bodies were collected from Chengdu, Dazhou, Guangan, Leshan, and Meishan in Sichuan Province, China, placed in sterilized plastic bags, and transported to the laboratory for analysis. The procedure for obtaining axenic cultures, as described by Chomnunti et al. (2014), involved isolating pure cultures, transferring them to PDA slants for 10 days of cultivation, and subsequently storing them at 4 °C. The specimens were deposited in the Herbarium of Sichuan Agricultural University (SICAU), Chengdu, China, while the strains were stored in the Culture Collection of Sichuan Agricultural University (SICAUCC), China.

### ﻿Morphological observations

Fruiting bodies were observed and photographed using a dissecting microscope NVTGG (Shanghai Advanced Photoelectric Technology Co. Ltd., Shanghai, China) fitted with a VS-800C micro-digital camera (Shenzhen Weishen Times Technology Co. Ltd., Shenzhen, China). The dimensions of conidiomata, paraphyses, conidiophores, conidiogenous cells, and conidia were measured from field samples and photographed using an Olympus BX43 compound microscope equipped with an Olympus DP22 digital camera, in conjunction with ACDSee v3.1 software. Measurements were made using Tarosoft® Image Frame Work v.0.9.7 (Tarosoft (R), Nontha Buri, Thailand). Lactophenol cotton blue reagent was used to observe the conidiogenous structures and determine the number of septa. To observe and document the color and texture of the colonies, fresh plates were prepared from purified colonies and incubated at 25 °C for 1 to 3 weeks.

### ﻿DNA extraction, amplification, and sequencing

Total genomic DNA was extracted from mycelia grown on PDA or single fruiting bodies using the Plant Genomic DNA extraction kit (Tiangen, China). Primer pairs ITS5 and ITS4 (White et al. 1990), NS1 and NS4 (White et al. 1990), LR0R and LR5 (Vilgalys and Hester 1990), EF1-983F and EF1-2218R (Rehner and Buckley 2005), RPB1-Ac and RPB1-Cr (Lombard et al. 2015), fRPB2-5F and fRPB2-7cR (Liu et al. 2000), and ACT512F/ACT-783R (Carbone and Kohn 1999) were used to amplify the internal transcribed spacer (ITS), the partial small subunit nuclear rDNA (SSU), the nuclear ribosomal large subunit (LSU), the translation elongation factor *tef*1-α, the largest subunit of RNA polymerase II (*rpb*1), the second largest subunit of RNA polymerase II (*rpb*2), and the actin gene (act), respectively. Additionally, for *Cladosporium* species, EF1728F and EF2 (Lombard et al. 2015) were used to amplify *tef*1-α.

Polymerase chain reaction (PCR) was performed in a 25 μL reaction mixture containing 22 μL Master Mix (Beijing TsingKe Biotech Co. Ltd., Beijing, China), 1 μL DNA template (10–30 ng/μL), and 1 μL of each primer (120–150 ng/μL). The amplification reactions were performed as described by Lombard et al. (2015), Dai et al. (2016), and Wang et al. (2022a). PCR products were sequenced at TsingKe Biological Technology Co. Ltd., Chengdu, China. The newly generated sequences were deposited in GenBank.

### ﻿Phylogenetic analyses

Phylogenetic analyses were conducted using sequences from *Cladosporium* (ITS, *act*, and *tef*1-α), *Moelleriella* (LSU, *rpb*1, and *tef*1-α), and *Podonectria* (ITS, LSU, SSU, *tef*1-α, and *rpb*2). Multigene sequences from various species (see Tables 1–3) were obtained from GenBank, along with additional sequences generated in this study. DNA alignments were carried out using the MAFFT v.7.429 online service (Katoh et al. 2019), and ambiguous regions were excluded using BioEdit version 7.0.5.3 (Hall 1999). Multigene sequences were concatenated using Mesquite software (Maddison and Maddison 2019). Multigene phylogenetic analyses were conducted using maximum likelihood (ML) and Bayesian inference (BI) methods. The optimal nucleotide substitution model was identified with MrModeltest v.2.2 (Nylander 2004).

**Table 1.** Voucher information and GenBank accession numbers of the taxa used in the *Cladosporium*.

Species	Voucher information	ITS	act	tef1-α	References
*Cladosporium acalyphae*	CBS 125982 ^T^	HM147994	HM148481	HM148235	Bensch et al. (2010)
*C. alboflavescens*	CBS 140690 ^T^	LN834420	LN834604	LN834516	Sandoval-Denis et al. (2016)
*C. angulosum*	CBS 140692 ^T^	LN834425	LN834609	LN834521	Sandoval-Denis et al. (2016)
*C. angustisporum*	CBS 125983 ^T^	HM147995	HM148482	HM148236	Bensch et al. (2010)
*C. angustiterminale*	CBS 140480 ^T^	KT600379	KT600575	KT600476	Bensch et al. (2015)
*C. anthropophilum*	CBS 140685 ^T^	LN834437	LN834621	LN834533	Sandoval-Denis et al. (2016)
*C. arenosum*	CHFC-EA 566 ^T^	MN879328	MN890008	MN890011	Crous et al. (2020a)
*C. armandiae*	CBS 153756 ^T^	PQ066521	PQ067354	PQ067355	Tan et al. (2024)
*C. asperulatum*	CBS 126340 ^T^	HM147998	HM148485	HM148239	Bensch et al. (2010)
*C. aulonemiae*	COAD 2269 ^T^	MZ318427	MT373119	MT680198	Costa et al. (2022)
*C. aulonemiae*	COAD 2270	MZ318428	MT373120	MT680199	Costa et al. (2022)
*C. australiense*	CBS 125984 ^T^	HM147999	HM148486	HM148240	Bensch et al. (2010)
*C. austroafricanum*	CBS 140481 ^T^	KT600381	KT600577	KT600478	Bensch et al. (2015)
*C. austrolitorale*	CBS 148321 ^T^	MN879327	MN890007	MN890010	Crous et al. (2021a)
*C. bambusicola*	COAD 2256 ^T^	MZ318433	MT373125	MT680204	Costa et al. (2022)
*C. bambusicola*	COAD 2565	OP535372	OP598124	OP676083	Costa et al. (2022)
*C. benschiae*	COAD 2263 ^T^	MZ318436	MT373128	MT680207	Costa et al. (2022)
*C. benschiae*	COAD 2265	MZ318437	MT373129	MT680208	Costa et al. (2022)
*C. bentivoglioae*	BRIP 74745a ^T^	OR947065	OR964960	OR964961	Tan and Shivas (2023a)
*C. brigadeirense*	COAD 2257 ^T^	MZ318435	MT373127	MT680206	Costa et al. (2022)
*C. caprifimosum*	FMR 16532 ^T^	LR813198	LR813205	LR813210	Iturrieta-González et al. (2021)
*C. cavernicola*	URM 8389 ^T^	MZ518829	MZ555746	MZ555733	Pereira et al. (2022)
*C. chalastosporoides*	CBS 125985	HM148001	HM148488	HM148242	Bensch et al. (2010)
*C. chasmanthicola*	CPC 21300 ^T^	KY646221	KY646224	KY646227	Marin-Felix et al. (2017)
*C. chlamydosporigenum*	AUMC 11340 ^T^	MN826919	OL514009		Moharram et al. (2022)
*C. chlamydosporiformans*	COAD 2571 ^T^	OP535374	OP598126	OP676085	Pereira et al. (2024)
*C. chlamydosporiformans*	COAD 2568	OP535378	OP598130	OP676089	Pereira et al. (2024)
*C. chusqueae*	COAD 2258 ^T^	MZ318430	MT373122	MT680201	Costa et al. (2022)
*C. chusqueae*	COAD 2261	MZ318431	MT373124	MT680203	Costa et al. (2022)
*C. chubutense*	CBS 124457 ^T^	FJ936158	FJ936165	FJ936161	Schubert et al. (2009)
*C. cladosporioides*	CBS 112388 ^T^	HM148003	HM148490	HM148244	Bensch et al. (2010)
*C. compactisporum*	AUMC 11366 ^T^	MN826822	OL514010		Moharram et al. (2022)
*C. colocasiae*	CBS 386.64 ^T^	HM148067	HM148555	HM148310	Bensch et al. (2010)
*C. colombiae*	CBS 274.80B ^T^	FJ936159	FJ936166	FJ936163	Schubert et al. (2009)
*C. congjiangense*	GUCC 21208.3 ^T^	OP852675	OP863094	OP859042	Yang et al. (2023)
*C. congjiangense*	GUCC 21208.5	OP852676	OP863095	OP859043	Yang et al. (2023)
*C. coprophilum*	FMR 16164 ^T^	LR813201	LR813207	LR813213	Iturrieta-González et al. (2021)
*C. corticola*	BRIP 74385a ^T^	OP256851	OP288997	OP288998	Crous et al. (2023)
*C. crousii*	CBS 140686 ^T^	LN834431	LN834615	LN834527	Sandoval-Denis et al. (2016)
*C. cucumerinum*	CBS 171.52 ^T^	HM148072	HM148561	HM148316	Bensch et al. (2010)
*C. delicatulum*	CBS 126344	HM148081	HM148570	HM148325	Bensch et al. (2010)
*C. devikae*	BRIP 72278a ^T^	MZ303808	MZ344212	MZ344193	Prasannath et al. (2021a, 2021b)
*C. diamantinense*	COAD 3108 ^T^	ON062328	ON141933	ON982817	Dutra et al. (2023)
*C. endoviticola*	JZBH 390018 ^T^	MN654960	MN984220	MN984228	Manawasinghe et al. (2020)
*C. eucommiae*	GUCC 401.1 ^T^	OL587465	OL519775	OL504966	Wang et al. (2022b)
*C. eucommiae*	GUCC 401.9	ON334729	ON383337		Wang et al. (2022b)
*C. europaeum*	CBS 134914 ^T^	HM148056	HM148543	HM148298	Bensch et al. (2018)
*C. europaeum*	CBS 116744	HM148053	HM148540	HM148294	Bensch et al. (2018)
*C. exasperatum*	CBS 125986	HM148090	HM148579	HM148334	Bensch et al. (2010)
*C. exile*	CBS 125987	HM148091	HM148580	HM148335	Bensch et al. (2010)
*C. flabelliforme*	CBS 126345	HM148092	HM148581	HM148336	
*C. flavovirens*	CBS 140462 ^T^	LN834440	LN834624	LN834536	Sandoval-Denis et al. (2016)
*C. funiculosum*	CBS 122129 ^T^	HM148094	HM148583	HM148338	Bensch et al. (2010)
*C. fuscoviride*	FMR 16385 ^T^	LR813200	LR813206	LR813212	Iturrieta-González et al. (2021)
*C. gamsianum*	CBS 125989 ^T^	HM148095	HM148584	HM148339	Bensch et al. (2010)
*C. globisporum*	CBS 812.96 ^T^	HM148096	HM148585	HM148340	Crous et al. (2011)
*C. grevilleae*	CBS 114271 ^T^	JF770450	JF770473	JF770472	Crous et al. (2011)
*C. guizhouense*	GUCC 401.7 ^T^	OL579741	OL519780	OL504965	Wang et al. (2022b)
*C. guizhouense*	GUCC 401.8	ON334728	ON383338	ON383470	Wang et al. (2022b)
***C. guizhouense***	**SICAUCC 25-0057**	**PV156487**	**PV153466**	**PV153474**	**In this study**
***C. guizhouense***	**SICAUCC 25-0058**	**PV156488**	**PV153467**	**PV153475**	**In this study**
*C. hemileiicola*	COAD 2567 ^T^	OP535376	OP598128	OP676087	Pereira et al. (2024)
*C. hemileiicola*	COAD 3350	OP535381	OP598133	OP676092	Pereira et al. (2024)
*C. heteropogonicola*	BRIP 72465a ^T^	OL307932	OL332743	OL332742	Tan et al. (2022)
*C. hillianum*	CBS 125988 ^T^	HM148097	HM148586	HM148341	Bensch et al. (2010)
*C. inversicolor*	CBS 401.80 ^T^	HM148101	HM148590	HM148345	Bensch et al. (2010)
*C. ipereniae*	CBS 140483 ^T^	KT600394	KT600589	KT600491	Bensch et al. (2015)
*C. iranicum*	CBS 126346 ^T^	HM148110	HM148599	HM148354	Bensch et al. (2010)
*C. kaiyangense*	GUCC 21265.2 ^T^	OP852665	OP863097	OP859045	Yang et al. (2023)
*C. kenpeggii*	CPC 19248 ^T^	KY646222	KY646225	KY646228	Marin-Felix et al. (2017)
***C. kuwanaspidis***	**SICAUCC 25-0063 ^T^**	**PV156489**	**PV153468**	**PV153480**	**In this study**
***C. kuwanaspidis***	**SICAUCC 25-0064**	**PV156490**	**PV153469**	**PV153481**	**In this study**
*C. lagenariiforme*	SFC20230103-M23 ^T^	OQ186119	OQ185167	OQ185128	Lee et al. (2023)
*C. lentulum*	FMR 16288 ^T^	LR813203	LR813209	LR813215	Iturrieta-González et al. (2021)
*C. licheniphilum*	CBS 125990	HM148111	HM148600	HM148355	Bensch et al. (2010)
*C. longicatenatum*	CBS 140485	KT600403	KT600598	KT600500	Bensch et al. (2015)
*C. longissimum*	CBS 300.96	DQ780352	EF101385	EU570259	
*C. lycoperdinum*	CBS 574.78C	HM148115	HM148604	HM148359	Prasannath et al. (2021a, 2021b)
*C. macadamiae*	BRIP 72269a ^T^	MZ303810	MZ344214	MZ344195	Jayasiri et al. (2019)
*C. magnoliigena*	MFLUCC 18-1559 ^T^	MK347813		MK340864	Lee et al. (2023)
*C. maltirimosum*	SFC20230103-M51 ^T^	OQ186147	OQ185195	OQ185155	Lee et al. (2023)
*C. marinum*	SFC20230103-M33 ^T^	OQ186129	OQ185177	OQ185137	Bensch et al. (2015)
*C. montecillanum*	CBS 140486 ^T^	KT600406	KT600602	KT600504	Bensch et al. (2010)
*C. myrtacearum*	CBS 126350 ^T^	HM148117	HM148606	HM148361	Yang et al. (2023)
*C. nayongense*	GUCC 21260.3 ^T^	OP852669	OP863106	OP859054	Zimowska et al. (2021)
*C. neapolitanum*	MgPo1 ^T^	MK387890	MK416051	MK416094	Bensch et al. (2018)
*C. needhamense*	CBS 143359 ^T^	MF473142	MF473991	MF473570	Bensch et al. (2018)
*C. neerlandicum*	CBS 143360 ^T^	KP701887	KP702010	KP701764	Bensch et al. (2018)
*C. neopsychrotolerans*	CGMCC 3.18031 ^T^	KX938383	KX938366	KX938400	Bensch et al. (2010)
*C. oxysporum*	CBS 125991	HM148118	HM148607	HM148362	Bensch et al. (2010)
*C. paracladosporioides*	CBS 171.54 ^T^	HM148120	HM148609	HM148364	Bensch et al. (2015)
*C. parapenidielloides*	CBS 140487 ^T^	KT600410	KT600606	KT600508	Rosado et al. (2019)
*C. passiflorae*	COAD 2135 ^T^	MH682175	MH729795	MH724943	Rosado et al. (2019)
*C. passifloricola*	COAD 2140 ^T^		MH729800	MH724948	Bensch et al. (2010)
*C. perangustum*	CBS 125996 ^T^	HM148121	HM148610	HM148365	Bensch et al. (2018)
*C. perangustum*	CBS 126365	HM148123	HM148612	HM148367	Bensch et al. (2018)
*C. perangustum*	CPC 11663	HM148128	HM148617	HM148372	Sandoval-Denis et al. (2016)
*C. perangustum*	CPC 13870	HM148142	HM148631	HM148386	Bensch et al. (2010)
*C. perangustum*	FMR 13321	LN834380	LN834564	LN834476	Sandoval-Denis et al. (2016)
*C. pernambucoense*	URM 8390 ^T^	MZ518830	MZ555747	MZ555734	Pereira et al. (2022)
*C. pernambucoense*	URM 8391	MZ518828	MZ555745	MZ555732	Pereira et al. (2022)
*C. phaenocomae*	CBS 128769	JF499837	JF499881	JF499875	Crous and Groenewald (2011)
*C. phyllactiniicola*	CBS 126352	HM148150	HM148639	HM148394	
*C. phyllophilum*	CBS 125992 ^T^	HM148154	HM148643	HM148398	Bensch et al. (2010)
*C. pini-ponderosae*	CBS 124456 ^T^	FJ936160	FJ936167	FJ936164	Schubert et al. (2009)
*C. polonicum*	MgPo1 ^T^	MK387894	MK416055	MK416098	Zimowska et al. (2021)
*C. proteacearum*	BRIP 72301a ^T^	MZ303809	MZ344213	MZ344194	Prasannath et al. (2021a, 2021b)
*C. pruni-salicinae*	GUCC 21206.1 ^T^	OP852683	OP863092	OP859041	Yang et al. (2023)
*C. pseudocladosporioides*	CBS 125993 ^T^	HM148158	HM148647	HM148402	Bensch et al. (2010)
*C. pseudochalastoporoides*	CBS 140490 ^T^	KT600415	KT600611	KT600513	Bensch et al. (2015)
*C. pseudotenuissimum*	COAD 2266 ^T^	MZ318439	MT373132	MT680211	Costa et al. (2022)
*C. punicae*	GUCC 21271.5 ^T^	OP852672	OP863108	OP859056	Yang et al. (2023)
*C. puris*	COAD 2487 ^T^	MK253337	MK249980	MK293777	Freitas et al. (2021)
*C. queenslandicum*	BRIP 72447a ^T^	OL307928	OL332736	OL332735	Tan et al. (2022)
*C. queenslandicum*	BRIP 72452a	OL307929	OL332738	OL332737	Tan et al. (2022)
*C. queenslandicum*	BRIP 72455a	OL307931	OL332741	OL332740	Tan et al. (2022)
*C. rectoides*	CBS 125994 ^T^	HM148193	HM148683	HM148438	Bensch et al. (2010)
*C. ribis*	GUCC 21244.1 ^T^	OP852666	OP863098	OP859046	Yang et al. (2023)
*C. rubrum*	CMG 28 ^T^	MN053018	MN066639	MN066644	Vicente et al. (2021)
*C. rugulovarians*	CBS 140495 ^T^	KT600459	KT600656	KT600558	Bensch et al. (2015)
*C. ruthsangerae*	BRIP 75808a ^T^	OR290122	OR335736	OR335741	Tan and Shivas (2023b)
*C. scabrellum*	CBS 126358 ^T^	HM148195	HM148685	HM148440	Bensch et al. (2010)
*C. setoides*	COAD 3470 ^T^	OP535379	OP598131	OP676090	Pereira et al. (2024)
*C. setoides*	COAD 2576	OP535380	OP598132	OP676091	Pereira et al. (2024)
*C. silenes*	CBS 109082 ^T^	EF679354	EF679506	EF679429	Schubert et al. (2007)
*C. sinuatum*	CGMCC 3.18096 ^T^	KX938385	KX938368	KX938402	Bensch et al. (2018)
*C. speluncae*	COAD 3116 ^T^	ON062329	ON141934	ON982818	Dutra et al. (2023)
*C. splattiae*	BRIP 75807a	OR290120	OR335734	OR335739	Tan and Shivas (2023b)
*C. sphaerospermum*	CBS 193.54	DQ780343	EF101380	EU570261	Crous et al. (2021b)
*C. stipagrostidicola*	CBS 146978 ^T^	MZ064420	MZ078146	MZ078223	Bensch et al. (2010)
*C. subuliforme*	CBS 126500 ^T^	HM148196	HM148686	HM148441	Bensch et al. (2010)
*C. tenuissimum*	CBS 125995 ^T^	HM148197	HM148687	HM148442	Bensch et al. (2018)
*C. tianshanense*	CGMCC 3.18033 ^T^	KX938381	KX938364	KX938398	Braun et al. (2003); Bensch et al. (2010)
*C. uredinicola*	ACC 46649	AY251071	HM148712	HM148467	Bensch et al. (2018)
*C. uwebraunianum*	CBS 143365 ^T^	MF473306	MF474156	MF473729	Bensch et al. (2010)
*C. varians*	CBS 126362 ^T^	HM148224	HM148715	HM148470	Bensch et al. (2010)
*C. verrucocladosporioides*	CBS 126363 ^T^	HM148226	HM148717	HM148472	Bensch et al. (2018)
*C. vicinum*	CBS 143366 ^T^	MF473311	MF474161	MF473734	Bensch et al. (2018)
*C. vignae*	CBS 121.25	HM148227	HM148718	HM148473	Marin-Felix et al. (2017)
*C. welwitschiicola*	CPC 18648 ^T^	KY646223	KY646226	KY646229	Yang et al. (2023)
*C. wenganense*	GUCC 21220.1 ^T^	OP852682	OP863101	OP859049	Bensch et al. (2018)
*C. westerdijkiae*	CBS 113746 ^T^	HM148061	HM148548	HM148303	Sandoval-Denis et al. (2016)
*C. xanthochromaticum*	CBS 140691 ^T^	LN834415	LN834599	LN834511	Bensch et al. (2010)
*C. xylophilum*	CBS 125997 ^T^	HM148230	HM148721	HM148476	Xu et al. (2021b)
*C. yunnanensis*	KUN-HKAS 121704 ^T^	OK338502	OL466937	OL825680	Zalar et al. (2007); Dugan et al. (2008)
*C. longissimum*	CBS 300.96 ^T^	DQ780352	EF101385	EU570259	Zalar et al. (2007); Dugan et al. (2008)

^T^ Type material. New species were shown in bold.

**Table 2.** Voucher information and GenBank accession numbers of the taxa used in the *Moelleriella*.

Species	Voucher information	LSU	rpb1	tef1-α	References
*Hypocrella citrina*	P.C. 606	EU392556	EU392640	EU392694	Chaverri et al. (2008)
*H. citrina*	P.C. 597	AY986905	AY986930	DQ000331	Chaverri et al. (2005a)
*H. discoidea*	BCC2097		AY986945	DQ000346	Chaverri et al. (2005a)
*H. discoidea*	I93-901d	EU392567	EU392646	EU392700	Chaverri et al. (2008)
*H. disciformis*	P.C. 655	EU392560	EU392643	EU392697	Chaverri et al. (2008)
*H. disciformis*	P.C. 676	EU392566	EU392645	EU392699	Chaverri et al. (2008)
*H. hirsuta*	P.C. 436.2	AY986922	AY986949	DQ000350	Chaverri et al. (2005a)
*H. hirsuta*	P.C. 543	EU392569	EU392648	EU392702	Chaverri et al. (2008)
*H. viridans*	P.C. 635	EU392572	EU392651	EU392705	Chaverri et al. (2008)
*H. viridans*	I89-490	EU392570	EU392649	EU392703	Chaverri et al. (2008)
*Moelleriella africana*	P.C. 736	AY986917	AY986943	DQ000344	Chaverri et al. (2005a)
*M. alba*	BCC49492	JQ269645	KX254424	JQ256905	Mongkolsamrit et al. (2015)
*M. alba*	BCC49409 ^T^	JQ269646	KX254423	JQ256906	Mongkolsamrit et al. (2015)
*M. basicystis*	P.C. 374	AY986903	AY986928	DQ000329	Chaverri et al. (2005a)
*M. basicystis*	F183147		EU392577	EU392653	Chaverri et al. (2008)
***M. boehmeriae***	**SICAUCC 25-0061 ^T^**	**PV124784**	**PV153478**	**PV153492**	**In this study**
***M. boehmeriae***	**SICAUCC 25-0062**	**PV124785**	**PV153479**	**PV153493**	**In this study**
*M. boliviensis*	P.C. 603	AY986923	AY986950	DQ000351	Chaverri et al. (2005a)
*M. chiangmaiensis*	BCC18029 ^T^		MT659360	MW091560	Khonsanit et al. (2021)
*M. chiangmaiensis*	BBH33051	MT659362	MT672277	MT672269	Khonsanit et al. (2021)
*M. chiangmaiensis*	BCC60941	MT659361	MT672278	MT672270	Khonsanit et al. (2021)
*M. chumphoensis*	BCC47575	JQ269648	KX254422	JQ256908	Mongkolsamrit et al. (2015)
*M. chumphoensis*	BCC47574 ^T^	JQ269647	KX254421	JQ256907	Mongkolsamrit et al. (2015)
***M. cinnamomum***	**SICAUCC 25-0067 ^T^**	**PV124788**	**PV153484**	**PV153494**	**In this study**
***M. cinnamomum***	**SICAUCC 25-0068**	**PV124789**	**PV153485**	**PV153495**	**In this study**
***M. citrus***	**SICAUCC 25-0059 ^T^**	**PV124782**	**PV153476**	**PV153490**	**In this study**
***M. citrus***	**SICAUCC 25-0060**	**PV124783**	**PV153477**	**PV153491**	**In this study**
*M. disjuncta*	J.B. 205		EU392578	EU392654	Chaverri et al. (2008)
*M. epiphylla*	P.C. 545	EU392585	EU392660	EU392711	Chaverri et al. (2008)
*M. epiphylla*	I93-813	EU392583	EU392656	EU392707	Chaverri et al. (2008)
***M. eucalypti***	**SICAU 25-0072 ^T^**	**PV124778**	**PV153470**	**PV153486**	**In this study**
***M. eucalypti***	**SICAU 25-0073**	**PV124779**	**PV153471**	**PV153487**	**In this study**
***M. eucalypti***	**SICAU 25-0074**	**PV124780**	**PV153472**	**PV153488**	**In this study**
***M. eucalypti***	**SICAU 25-0075**	**PV124781**	**PV153473**	**PV153489**	**In this study**
*M. evansii*	P.C. 627 ^T^	AY986916	AY986942	DQ000343	Chaverri et al. (2005a)
*M. flava*	BCC60924 ^T^	KF951146	KX254430	MT672271	Khonsanit et al. (2021)
*M. flava*	BCC60925	KF951147	KX254431	MT672272	Khonsanit et al. (2021)
*M. flava*	BCC60929	KX298238	KX254432	MT672273	Khonsanit et al. (2021)
*M. flava*	BCC60930		KX298237	KX254429	Khonsanit et al. (2021)
*M. globostromata*	YFCC 22109275 ^T^	OR828408	OR831942	OR831952	Wang et al. (2025)
*M. globostromata*	YHH 221010	OR828403	OR831940	OR831950	Wang et al. (2025)
*M. gracilispora*	CGMCC 3.18989 ^T^	KC964202	KC964191	KC964179	Yuan et al. (2020)
*M. gracilispora*	CGMCC 3.18990	KC964203	KC964192	KC964180	Yuan et al. (2020)
*M. hainanensis*	YHH 2303020	OR828400	OR831938	OR831948	Wang et al. (2025)
*M. hainanensis*	YFCC 23039277 ^T^		OR831939	OR831949	Wang et al. (2025)
*M. insperata*	ARSEF 2396 ^T^	AY518374	DQ070029	EU392713	Chaverri et al. (2008)
*M. jinghongensis*	YFCC 23089312 ^T^		OR854253	OR837093	Wang et al. (2025)
*M. jinghongensis*	YHH 2308025	OR828411	OR854254	OR837094	Wang et al. (2025)
*M. jinuoana*	YHH MJBP2309031 ^T^	PP178643	PP776170	PP776160	Wang et al. (2024)
*M. jinuoana*	YHH MJBP2309032	PP178644	PP776171	PP776161	Wang et al. (2024)
*M. jinuoana*	YFCC MJBP23099451	PP178645	PP776172	PP776162	Wang et al. (2024)
*M. kanchanaburiensis*	BCC75979	MT659363	MT672279	MT843900	Khonsanit et al. (2021)
*M. kanchanaburiensis*	BCC75980	MT659364	MT672280	MT843901	Khonsanit et al. (2021)
*M. kanchanaburiensis*	BCC75981 ^T^		MT659365	MT672281	Khonsanit et al. (2021)
*M. libera*	P.C. 444	EU392591	EU392662	EU392714	Chaverri et al. (2008)
*M. libera*	P.C. 445	AY986900	AY986925	DQ000326	Chaverri et al. (2005a)
*M. longzhuensis*	YHH MLFSL2310012 ^T^	PP178646	PP776173	PP776163	Wang et al. (2024)
*M. longzhuensis*	YHH MLFSL2310013	PP178647	PP776174	PP776164	Wang et al. (2024)
*M. longzhuensis*	YFCC-MLFSL23109453		PP776175	PP776165	Wang et al. (2024)
*M. macrostroma*	P.C. 605 ^T^	AY986919	AY986946	DQ000347	Chaverri et al. (2005a)
*M. macrostroma*	J.B. 115	AY986920	AY986947	DQ000348	Chaverri et al. (2005a)
*M. madidiensis*	P.C. 569	AY986915	AY986941	DQ000342	Chaverri et al. (2005a)
*M. madidiensis*	P.C. 594	EU392595	EU392666	EU392718	Chaverri et al. (2008)
*M. mollii*	I93-901a	EU392599	EU392667	EU392719	Chaverri et al. (2008)
*M. mollii*	I93-901c	EU392600	EU392668	EU392720	Chaverri et al. (2008)
*M. multiperitheciata*	YFCC 23089307 ^T^	OR828407	OR832085	OR837089	Wang et al. (2025)
*M. multiperitheciata*	YFCC 22109308	OR828406	OR832086	OR837090	Wang et al. (2025)
*M. nanensis*	BCC66303 ^T^	KX298236	KX254427	MW085940	Khonsanit et al. (2021)
*M. nanensis*	BCC66305	MW080317	KX254428	MW085941	Khonsanit et al. (2021)
*M. nivea*	BCC60891 ^T^	MW080318	MT672282	MW085942	Khonsanit et al. (2021)
*M. nivea*	BCC58543	MT659366	MT672283	MT672274	Khonsanit et al. (2021)
*M. nivea*	BCC58544	MT659367	MT672284	MT843898	Khonsanit et al. (2021)
*M. ochracea*	IE 1308	EU392601	EU392669	EU392721	Chaverri et al. (2008)
*M. ochracea*	P.C. 648	EU392605	EU392671	EU392723	Chaverri et al. (2008)
*M. oxystoma*	MDYS2010-08	KC995287	KC964188	KC964176	Yang et al. (2023)
*M. oxystoma*	BCC 9482	DQ377986	DQ384957	DQ385013	Yang et al. (2023)
*M. phukhiaoensis*	BCC19769 ^T^		KT880502	KT880506	Li et al. (2016)
*M. phukhiaoensis*	BCC19773		KT880503	KT880507	Li et al. (2016)
*M. phyllogena*	P.C. 555	EU392610	EU392674	EU392726	Chaverri et al. (2008)
*M. phyllogena*	J.B. 130	EU392608	EU392672	EU392724	Chaverri et al. (2008)
*M. pongdueatensis*	BCC31787 ^T^	KT880500	KX254433	KT880504	Li et al. (2016)
*M. pongdueatensis*	BCC31788	KT880501	KX254434	KT880505	Li et al. (2016)
*M. pseudothanathonensis*	YFCC 22099302 ^T^		OR842379	OR837103	Wang et al. (2025)
*M. pseudothanathonensis*	YHH 2209005	OR828404	OR842382	OR837106	Wang et al. (2025)
*M. puerensis*	YFCC 8615 ^T^	MW786748	MW815596	MW815595	Wang et al. (2022c)
*M. puerensis*	YFCC 8625	MW786749	MW815597	MW815593	Wang et al. (2022c)
*M. puertoricoensis*	BCC88320 ^T^	MN954683	MN944389		Crous et al. (2020b)
*M. puertoricoensis*	BBC88321	MN954684	MN944390		Crous et al. (2020b)
*M. puertoricoensis*	BCC88322	MN954682	MN944391		Crous et al. (2020b)
*M. puwenensis*	YHH 2308029 ^T^	OR828412	OR854257	OR831953	Wang et al. (2025)
*M. puwenensis*	YHH 2308030	OR828413	OR854258	OR831954	Wang et al. (2025)
*M. qionzhongensis*	YHH 2303021	OR828399	OR831936	OR831946	Wang et al. (2025)
*M. qionzhongensis*	YFCC 23039306 ^T^		OR831937	OR831947	Wang et al. (2025)
*M. raciborskii*	Afr 28	DQ070113	EU392675	EU392727	Chaverri et al. (2008)
*M. raciborskii*	I93-901b	EU392611	EU392676	EU392728	Chaverri et al. (2008)
*M. reineckeana*	BCC1713		DQ384968	DQ385008	Mongkolsamrit et al. (2015)
*M. reineckeana*	BCC1765		DQ384958	DQ385010	Mongkolsamrit et al. (2015)
*M. rhombispora*	P.C. 467	AY986908	AY986933	DQ000334	Chaverri et al. (2005a)
*M. rhombispora*	P.C. 696	EU392618	EU392680	EU392732	Chaverri et al. (2008)
*M. schizostachyi*	CBS 100067	AY986921	AY986948	DQ000349	Chaverri et al. (2005a)
*M. simaoensis*	YFCC 9249 ^T^	OQ621809	OQ623181	OQ616917	Yang et al. (2023)
*M. simaoensis*	YHH 2210016	OQ621808	OQ623180	OQ616916	Yang et al. (2023)
*M. sinensis*	CGMCC 3.18911 ^T^	MK412091		MK412101	Chen et al. (2020)
*M. sinensis*	BCC69128	KX298234	KX254425	MT843899	Khonsanit et al. (2021)
*M. sloaneae*	I94-920	EU392621	EU392682	EU392734	Chaverri et al. (2008)
*M. sloaneae*	I94-922c	EU392622	EU392683	EU392735	Chaverri et al. (2008)
*M. turbinata*	P.C. 678	EU392627	EU392687	EU392739	Chaverri et al. (2008)
*M. turbinata*	IMI 352838	EU392625	EU392685	EU392737	Chaverri et al. (2008)
*M. umbospora*	P.C. 457	AY986904	AY986929	DQ000330	Chaverri et al. (2005a)
*M. umbospora*	P.C. 461 ^T^	EU392628	EU392688	EU392740	Chaverri et al. (2008)
*M. yuanyangensis*	YFCC 23039314 ^T^		OR831945	OR837097	Wang et al. (2025)
*M. yuanyangensis*	YHH 2209001		OR831944	OR837098	Wang et al. (2025)
*M. yunnanensis*	YFCC 23089310 ^T^		OR832093	OR837102	Wang et al. (2025)
*M. yunnanensis*	YHH 2308001	OR828416	OR832091	OR837100	Wang et al. (2025)
*M. zhongdongii*	P.C. 504	EU392631	EU392689	EU392741	Chaverri et al. (2008)
*M. zhongdongii*	P.C. 549	EU392632	EU392690	EU392742	Chaverri et al. (2008)
*Purpureocillium lilacinum*	CBS 284.36 ^T^	FR775484	EF468792	EF468898	Sung et al. (2007)
*P. lilacinum*	CBS 431.87	EF468844	EF468791	EF468897	Sung et al. (2007)
*Regiocrella camerunensis*	CUP 67512	DQ118735	DQ118743	DQ127234	Chaverri et al. (2005b)
*R. sinensis*	CUP CH-2640	DQ118736	DQ118744	DQ127235	Chaverri et al. (2005b)
*Samuelsia chalalensis*	P.C. 560 ^T^	EU392637	EU392691	EU392743	Chaverri et al. (2008)
*S. geonomis*	P.C. 614	EU392638	EU392692	EU392744	Chaverri et al. (2008)
*S. mundiveteris*	BCC40021		GU552152	GU552145	Mongkolsamrit et al. (2011a)
*S. mundiveteris*	BCC40022		GU552153	GU552146	Mongkolsamrit et al. (2011a)
*S. rufobrunnea*	P.C. 613	AY986918	AY986944	DQ000345	Chaverri et al. (2005a); Mongkolsamrit et al. (2011a)

^T^ Type material. New species were shown in bold.

**Table 3.** Voucher information and GenBank accession numbers of the taxa used in the *Podonectria*.

Species	Voucher information	ITS	LSU	SSU	tef1-α	rpb2	References
*Alloleptosphaeria clematidis*	MFLUCC 17-2071 ^T^	MT310604	MT214557	MT226674	MT394736	MT394685	Phukhamsakda et al. (2020)
*Alternaria aconidiophora*	CBS 145419 ^T^	LR133931			LR133968	LR133967	Woudenberg et al. (2013)
*Alt. alternata*	CBS 916.96 ^T^	AF347031	DQ678082	KC584507	KC584634	KC584375	Marin-Felix et al. (2019)
*Alt. dactylidicola*	MFLUCC 15-0466 ^T^	KY703616	KY703617	KY703618		KY750720	Thambugala et al. (2017)
*Astragalicola amorpha*	CBS 142999 ^T^	MF795753	MF795753		MF795842	MF795795	Jaklitsch et al. (2018)
*A. vasilyevae*	MFLUCC 17-0832 ^T^	MG828870	MG828986	MG829098	MG829193	MG829248	Wanasinghe et al. (2018)
*Bambusicola bambusae*	MFLUCC 11-0614 ^T^	JX442031	JX442035	JX442039	KP761722	KP761718	Dai et al. (2012, 2015)
*B. didymospora*	MFLUCC 10-0557 ^T^	KU940116	KU863105	KU872110	KU940188	KU940163	Dai et al. (2016)
*B. dimorpha*	MFLUCC 13-0282 ^T^	KY026582	KY000661	KY038354		KY056663	Thambugala et al. (2017)
*B. irregularispora*	MFLUCC 11-0437 ^T^	JX442032	JX442036	JX442040	KP761723	KP761719	Dai et al. (2012, 2015)
*B. loculata*	MFLUCC 13-0856 ^T^	KP761732	KP761729	KP761735	KP761724	KP761715	Dai et al. (2015)
*B. massarinia*	MFLUCC 11-0389 ^T^	JX442033	JX442037	JX442041	KP761725	KP761716	Dai et al. (2012, 2015)
*B. pustulata*	MFLUCC 15-0190 ^T^	KU940118	KU863107	KU872112	KU940190	KU940165	Dai et al. (2016)
*B. sichuanensis*	SICAUCC 16-0002 ^T^	MK253473	MK253532	MK253528	MK262828	MK262830	Yang et al. (2019a)
*B. splendida*	MFLUCC 11-0439 ^T^	JX442034	JX442038	JX442042	KP761726	KP761717	Dai et al. (2012, 2015)
*B. subthailandica*	SICAU 16-0005 ^T^	MK253474	MK253533	MK253529	MK262829	MK262831	Yang et al. (2019a)
*B. thailandica*	MFLUCC 11-0147 ^T^	KU940119	KU863108	KU872113	KU940191	KU940166	Dai et al. (2016)
*B. triseptatispora*	MFLUCC 11-0166 ^T^	KU940120	KU863109			KU940167	Dai et al. (2016)
*Boeremia coffeae*	CBS 109183	GU237748	GU237943		KY484678	KT389566	Jayawardena et al. (2019)
*B. linicola*	CBS 116.76 ^T^	GU237754	GU237938		KY484705	KT389574	Jayawardena et al.
*B. opuli*	CGMCC 3.18354 ^T^	KY742045	KY742199			KY742133	(2019) Jayawardena et al. (2019)
*B. populi*	CBS 100167 ^T^	GU237707	GU237939		KY484706		Jayawardena et al. (2019)
*B. rhapontica*	CBS 113651 ^T^	KY484662			KY484713		Jayawardena et al. (2019)
*Coniothyrium chiangmaiense*	MFLUCC 16-0891 ^T^	KY568987	KY550384	KY550385		KY607015	Thambugala et al. (2017)
*C. sidae*	CBS 135108 ^T^	KF251149	KF251653		KF253109	KF252158	Quaedvlieg et al. (2013)
*C. telephii*	UTHSC:DI16-189	LT796830	LN907332			LT796990	Valenzuela-Lopez et al. (2017a)
*Cucurbitaria berberidis*	CBS 363.93	JF740191	GQ387606				de Gruyter et al. (2012)
*Decorospora gaudefroyi*	CBS 332.63	AF394541		AF394542			Inderbitzin et al. (2002)
*Didymella poaceicola*	MFLUCC 13-0212 ^T^	KX965726	KX954395			KX898364	Thambugala et al. (2017)
*Dothidotthia robiniae*	MFLUCC 16-1175 ^T^	MK751727	MK751817	MK751762	MK908017	MK920237	Senwanna et al. (2019)
*Epicoccum poaceicola*	MFLUCC 15-0448 ^T^	KX965727	KX954396			KX898365	Thambugala et al. (2017)
*E. thailandicum*	MFLUCC 16-0892 ^T^	KY703619	KY703620	KT454728			Thambugala et al. (2017)
*Leptosphaeria cichorium*	MFLUCC 14-1063 ^T^	KT454720	KT454712				Ariyawansa et al. (2015)
*Nothophoma chromolaenae*	MFLUCC 17-1443 ^T^	MT214364	MT214458	MT214410			Mapook et al. (2020)
*Ophiosimulans tanaceti*	MFLUCC 14-0525 ^T^	KU738890	KU73889	KU738892	MG520910		Tibpromma et al. (2016); Phookamsak et al. (2017)
*Palmiascoma gregariascomum*	MFLUCC 11-0175 ^T^	KP744452	KP744495	KP753958		KP998466	Liu et al. (2015)
*Parafenestella alpina*	CBS 145263 ^T^	MK356302	MK356302	MK357574	MK357530		Jaklitsch and Voglmayr (2019)
*P.raf. austriaca*	CBS 145262 ^T^	MK356304	MK356304	MK357576	MK357532		Jaklitsch and Voglmayr (2019)
*Paraophiobolus plantaginis*	MFLUCC 17-0245 ^T^	KY797641	KY815010	KY815012	MG520913		Phookamsak et al. (2017)
*Phaeosphaeria ampeli*	MFLUCC 18-1641 ^T^	MK503797	MK503808	MK503802	MK503814		Tennakoon et al. (2019)
*Podonectria coccicola*	PAaK	KU587798	KU519419				Dao et al. (2016)
*P. coccicola*	PUcS15	KU720533	KU519420				Dao et al. (2016)
*P. kuwanaspidis*	SICAUCC 21-0002 ^T^	MW484989	MW462900	MW462892	MW462112	MW462119	Xu et al. (2021)
*P. kuwanaspidis*	SICAUCC 21-0003	MW484990	MW462901	MW462893	MW462113	MW462120	Xu et al. (2021)
*P. kuwanaspidis*	SICAUCC 21-0007	MW484994	MW462905	MW462897	MW462116	MW462123	Xu et al. (2021)
***P. multiarmata***	**SICAUCC 25-0065 ^T^**	**PV156491**	**PV124786**	**PV135973**	**PV153482**	**PV153496**	**In this study**
***P. multiarmata***	**SICAUCC 25-0066**	**PV156492**	**PV124787**	**PV135974**	**PV153483**	**PV153497**	**In this study**
*P. novae-zelandiae*	PUcS14	KU720535	KU559551				Dao et al. (2016)
*P. novae-zelandiae*	PUcS13	KU720538	KU559548				Dao et al. (2016)
*P. novae-zelandiae*	PUcS12	KU720537	KU529802				Dao et al. (2016)
*P. novae-zelandiae*	PUcS11	KU720536	KU568479				Dao et al. (2016)
*P. novae-zelandiae*	SICAUCC 21-0004	MW484991	MW462902	MW462894	MW462114	MW462121	Xu et al. (2021)
*P. novae-zelandiae*	SICAUCC 21-0005	MW484992	MW462903	MW462895	MW462115	MW462122	Xu et al. (2021)
*P. sichuanensis*	SICAU 16-0003 ^T^	MK305903	MK296471	MK296467	MK313852	MK313855	Yang et al. (2019b)
*P. sichuanensis*	SICAUCC 21-0001	MW484988	MW462899	MW462891	MW462111	MW462118	Xu et al. (2021)
*Pseudoophiobolus galii*	MFLUCC 17-2257 ^T^	MG520947	MG520967	MG520989	MG520926		Phookamsak et al. (2017)
*Pseudopyrenochaeta lycopersici*	CBS 306.65 ^T^	NR_103581	EU754205			LT717680	Valenzuela-Lopez et al. (2017b)
*Pseudop. terrestris*	CBS 282.72 ^T^	LT623228	LT623216			LT623287	Valenzuela-Lopez et al. (2017b)
*Sclerenchymomyces clematidis*	MFLUCC 17-2180 ^T^	MT310605	MT214558	MT226675	MT394737	MT394686	Phukhamsakda et al. (2020)
*Seltsamia ulmi*	CBS 143002 ^T^	MF795794	MF795794	MF795794	MF795882	MF795836	Jaklitsch et al. (2018)
*Thyrostroma lycii*	MFLUCC 16-1170 ^T^	MK751734	MK751824	MK751769	MK908024	MK920241	Senwanna et al. (2019)
*Th. robiniae*	MFLUCC 18-1191 ^T^	MK751735	MK751825	MK751770	MK908025	MK920242	Senwanna et al. (2019)
*Tubeufia chiangmaiensis*	MFLUCC 11-0514 ^T^	KF301530	KF301538	KF301543	KF301557		Boonmee et al. (2014)
*Tu. javanica*	MFLUCC 12-0545 ^T^	KJ880034	KJ880036	KJ880035	KJ880037		Boonmee et al. (2014)

^T^ Type material. New species were shown in bold.

ML and BI analyses were performed using the CIPRES Science Gateway web server (Miller et al. 2010). For the ML analysis, RAxML-HPC2 on XSEDE (v.8.2.10) (Stamatakis 2014) was employed with the GTR+GAMMA substitution model and 1,000 bootstrap iterations. For BI analyses, the best-fit models were selected using MrModeltest v.2.2 for each dataset: *Cladosporium* (ITS: SYM+I+G, *act*: GTR+G, *tef*1-α: GTR+I+G), *Moelleriella* (LSU: GTR+I+G, *rpb*1: GTR+I+G, *tef*1-α: GTR+I+G), and *Podonectriaceae* (ITS: GTR+I+G, LSU: GTR+I+G, SSU: GTR+I+G, *tef*1-α: GTR+I+G, *rpb*2: GTR+I+G). The analyses were computed with six simultaneous Markov Chain Monte Carlo (MCMC) chains, run for 1,000,000 to 10,000,000 generations with a sampling frequency of every 100 generations. The burn-in fraction was set to 0.25, and the run was automatically terminated when the average standard deviation of split frequencies fell below 0.01.

Phylogenetic trees were visualized using FigTree v.1.4.3 (Rambaut and Drummond 2016) and further edited with Adobe Illustrator CS6 (Adobe Systems Inc., United States). Maximum likelihood bootstrap values (MLBS) of 60% or greater and Bayesian posterior probabilities (BIPP) of 0.95 or higher were indicated on the trees.

### ﻿Abbreviations

***act*** The actin gene

**BI** Bayesian inference

**bp** Base pair

**BIPP** Bayesian inference posterior probabilities

**DNA** Deoxyribonucleic acid

**ITS** internal transcribed spacer

**LSU** The nuclear ribosomal large subunit

**MEA** Malt extract agar medium

**ML** Maximum likelihood

**MLBS** Maximum likelihood bootstrap proportions

**PCR** Polymerase chain reaction

**PDA** Potato dextrose agar medium

***rpb*1** The largest subunits of RNA polymerase II

***rpb*2** The RNA polymerase II second largest subunit

**SICAU** The herbarium of Sichuan Agricultural University

**SICAUCC** The culture collection in Sichuan Agricultural University

**SNA** Synthetic low nutrient agar medium

**SSU** The partial small subunit nuclear rDNA

***tef*1-α** The translation elongation factor 1α

## ﻿Results

### ﻿Sequencing and phylogenetic analyses

Nucleotide BLAST results from GenBank indicated that our isolates were related to *Cladosporium*, *Moelleriella*, and *Podonectria*. To determine the phylogenetic relationships of our isolates within these genera, three phylogenetic analyses were conducted in this study.

### ﻿Phylogenetic analysis of the genus *Cladosporium*

**Figure 1.**
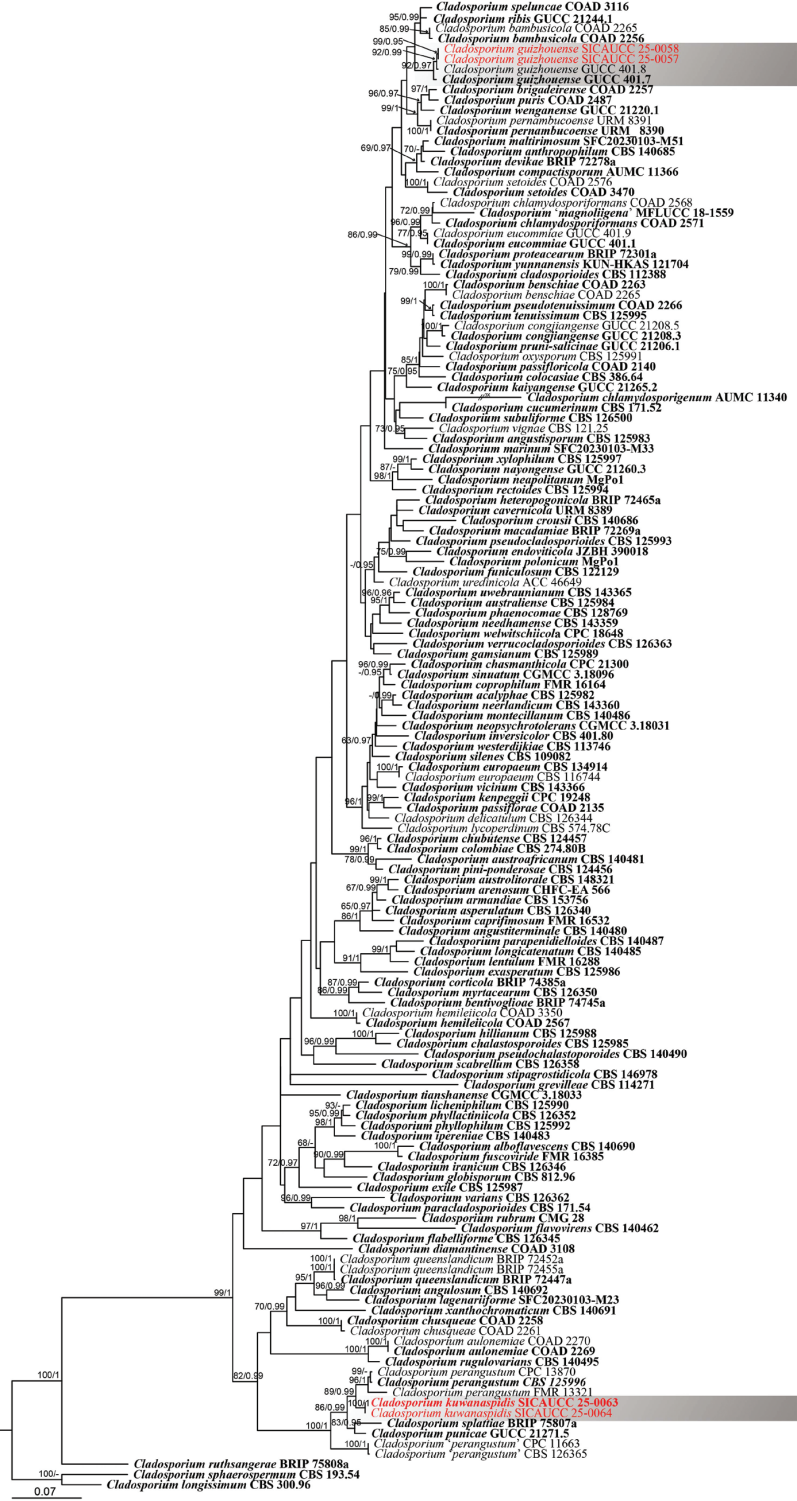
Phylogenetic tree based on maximum likelihood analysis of the combined *act*, ITS, and *tef*1-α sequence alignment dataset of the *Cladosporium cladosporioides* complex. Numbers above the nodes represent MLBS values (≥ 60%, left) and BIPP values (≥ 0.95, right), with lower values denoted as “–”. The tree is rooted to *C. sphaerospermum* (CBS 193.54) and *C. longissimum* (CBS 300.96). Ex-type strains are highlighted in bold, and isolates from the present study are shown in red.

An initial analysis of isolates from the three *Cladosporium* species complexes (*C. cladosporioides*, *C. herbarum*, and *C. sphaerospermum*) revealed that all *Cladosporium* isolates examined in this study belong to the *C. cladosporioides* species complex. The final alignment consisted of 122 taxa within the *C. cladosporioides* species complex, rooted with *C. sphaerospermum* (CBS 193.54) and *C. longissimum* (CBS 300.96) (*C. sphaerospermum* species complex) (Fig. 1). The alignment contained 2,008 characters (*act* = 1–273, ITS = 274–973, *tef*1-α = 974–2,008), including gaps. The best-scoring randomized accelerated maximum likelihood tree, with a final likelihood value of –20,491.871768, is presented. The matrix had 1,070 distinct alignment patterns, with 43.95% undetermined characters or gaps. The gamma distribution shape parameter α = 0.232201, and the tree length = 5.319454. The Bayesian analysis resulted in 200,002 trees after 10,000,000 generations, from which 150,002 were used for calculating posterior probabilities after the first 25% of trees, representing the burn-in phase, were discarded. Phylogenetic trees generated from ML and BI analyses were similar in overall topologies (Fig. 1). Within the *C. cladosporioides* species complex, our two isolates formed a clade with *C. guizhouense*, exhibiting strong bootstrap support values (92% MLBS/0.97 BIPP). Meanwhile, another two isolates clustered in a separate clade, distinct from known species within this complex, suggesting that they represent new species. *Cladosporium kuwanaspidis* sp. nov. (SICAUCC 25-0063, SICAUCC 25-0064) formed a monophyletic group closely related to *C. perangustum*.

### ﻿Phylogenetic analysis of the genus *Moelleriella*

**Figure 2.**
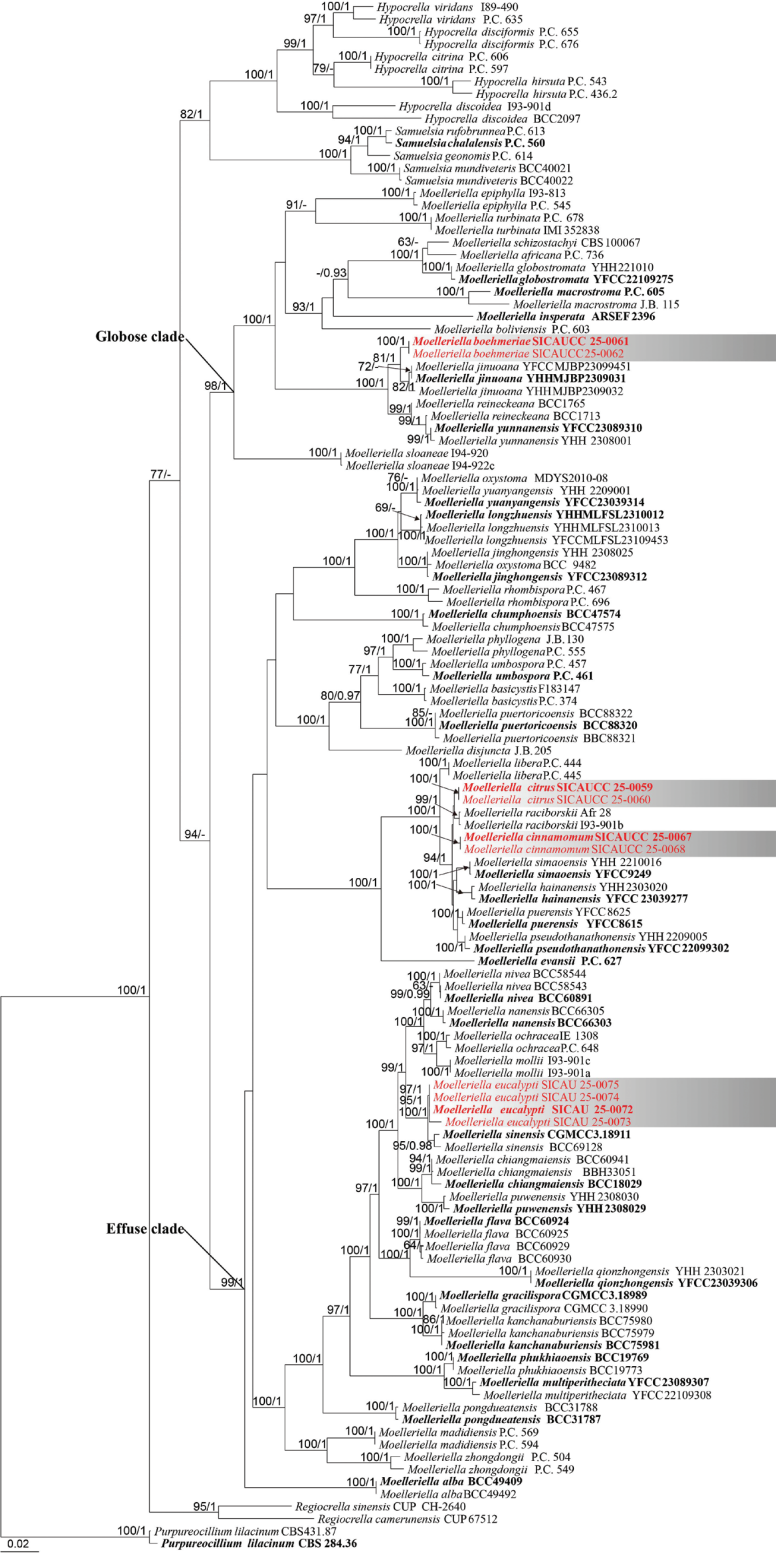
Phylogenetic tree based on maximum likelihood analysis of the combined LSU, *rpb*1, and *tef*1-α sequences within the *Clavicipitaceae*. Numbers above the nodes indicate MLBS values (≥ 60%, left) and BIPP values (≥ 0.95, right), with lower values denoted as “–”. The tree is rooted to *Purpureocillium lilacinum* (CBS 431.87, CBS 284.36). Ex-type strains are highlighted in bold, and isolates from the present study are shown in red.

A combined LSU–*rpb*2–*tef*1-α dataset, consisting of 62 taxa from the *Clavicipitaceae* and one outgroup taxon, *Purpureocillium lilacinum* (CBS 284.36, CBS 431.87) from the *Ophiocordycipitaceae*, was used for phylogenetic analyses (Fig. 2). The alignment contained 2,729 characters (LSU = 1–1,040, *rpb*1 = 1,041–1,788, *tef*1-α = 1,789–2,729), including gaps. The best-scoring tree with a final likelihood value of –27,989.959271 is presented. The matrix had 1,379 distinct alignment patterns, with 19.07% undetermined characters or gaps. The gamma distribution shape parameter α = 0.246493, and the tree length = 2.839149. The Bayesian analysis resulted in 31,182 trees after 50,000,000 generations, from which 23,388 were used for calculating posterior probabilities after the first 25% of trees, representing the burn-in phase, were discarded. Phylogenetic trees generated from ML and BI analyses were similar in overall topologies. Phylogeny from the combined sequence data analysis indicates that all the isolates belong to *Moelleriella* (Fig. 2). The isolates clustered in clades different from known species of *Moelleriella*, indicating that they are new species. *Moelleriella eucalypti* sp. nov. (SICAU 25-0072, SICAU 25-0073, SICAU 25-0074, SICAU 25-0075) formed a monophyletic group closely related to *M. sinensis*. *Moelleriella boehmeriae* sp. nov. (SICAUCC 25-0061 and SICAUCC 25-0062) formed a new clade closely related to *M. jinuoana*. Finally, *M. cinnamomum* sp. nov. (SICAUCC 25-0067, SICAUCC 25-0068) and *M. citrus* sp. nov. (SICAUCC 25-0059, SICAUCC 25-0060) formed a well-supported group containing *M. raciborskii*, *M. simaoensis*, *M. hainanensis*, *M. puerensis*, and *M. pseudothanathonensis*, but clustered in separate clades.

### ﻿Phylogenetic analysis of the genus *Podonectria*

**Figure 3.**
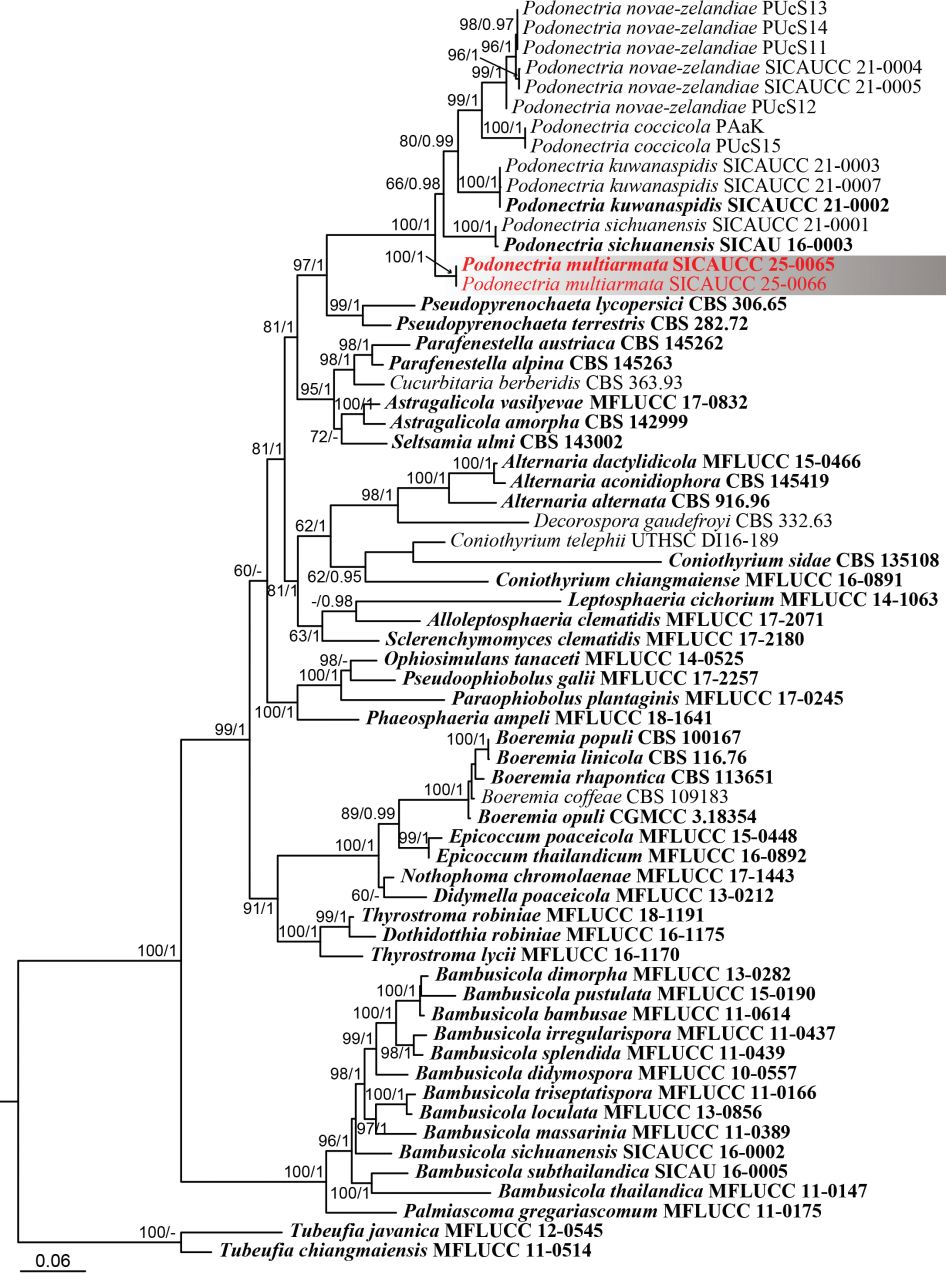
Phylogenetic tree based on maximum likelihood analysis of the combined ITS, LSU, SSU, *tef*1-α, and *rpb*2 sequences within the *Pleosporales*. Numbers above the nodes represent MLBS values (≥ 60%, left) and BIPP values (≥ 0.95, right), with lower values denoted as “–”. The tree is rooted to *Tubeufia javanica* (MFLUCC 12-0545) and *T. chiangmaiensis* (MFLUCC 11-0514). Ex-type strains are highlighted in bold, and isolates from the present study are shown in red.

To infer the relationships of the *Podonectria*-like taxa, a combined dataset of ITS, LSU, SSU, *tef*1-α, and *rpb*2 sequences was used to construct the phylogenetic tree. Phylogenetic analyses were conducted using a combined five-gene dataset comprising 52 taxa, with the tree rooted using *Tubeufia javanica* (MFLUCC 12-0545) and *T. chiangmaiensis* (MFLUCC 11-0514). The alignment contained 5,721 characters (ITS = 1–769, LSU = 770–1,744, *rpb*2 = 1,745–2,882, SSU = 2,883–4,301, *tef*1-α = 4,302–5,794), including gaps. The best-scoring tree with a final likelihood value of –41,343.562028 is presented. The matrix had 2,466 distinct alignment patterns, with 46.25% undetermined characters or gaps. The gamma distribution shape parameter α = 0.247235, and the tree length = 3.864868. The Bayesian analysis resulted in 4,602 trees after 1,000,000 generations, from which 3,452 were used for calculating posterior probabilities after the first 25% of trees, representing the burn-in phase, were discarded. Phylogenetic trees generated from ML and BI analyses were similar in overall topologies. The phylogeny derived from the combined sequence data analysis indicates that the novel strains form an independent lineage within the *Podonectriaceae* (Fig. 3). Based on the phylogenetic tree, our isolates clustered in a clade with species of *Podonectria*.

### ﻿Taxonomy

#### ﻿ Cladosporium kuwanaspidis

Taxon classificationAnimaliaCladosporialesCladosporiaceae

X.L. Xu & C.L. Yang sp. nov.

C57536B9-493B-5E7F-B279-114DAE7312BB

MB 858369

Fig. 4

##### Etymology.

In reference to the generic name for the associated scale insect (*Kuwanaspis howardi*).

##### Diagnosis.

Similar to *Cladosporium perangustum* in having conidiophores of comparable size, but *C. kuwanaspidis* differs by its unbranched conidiophores and larger ramoconidia.

##### Type.

**CHINA** • Sichuan Province, Meishan City, Hongya County. Infected scale insects (*Kuwanaspis howardi*) were found on the culms of bamboo (*Pleioblastus amarus* (Keng) P. C. Keng), 29°41.88'N, 103°14.04'E, alt. 540 m, 13 Mar. 2021, C.L. Yang, YCL202103004 (SICAU 25-0082 – holotype preserved in the Herbarium of Sichuan Agricultural University; living culture SICAUCC 25-0063 – ex-holotype stored in the Culture Collection in Sichuan Agricultural University).

##### Description.

Parasitic on scale insect from *Pleioblastus amarus* (*Poaceae*). **Sexual morph**: Not observed. **Asexual morph**: Hyphomycetous. Mycelium superficial and immersed, with abundant sporulation on the surface of scale insect. Conidiophores erect, fasciculate, usually macronematous, cylindrical, subnodulose or nodulose, geniculate, septate, unbranched, pale brown to brown, slightly roughened to verruculose, thick-walled, and pronounced loci, 45–120 × 3.5–6 μm. Conidiogenous cells terminal or intercalary, cylindrical, sometimes sinuous, proliferation sympodial, 14–25(–80) × 2.5–6.5 μm, conidiogenous loci at the apex (2–5) or in lateral shoulders (0–2). Ramoconidia olive to brown, septate or aseptate, ellipsoidal to subcylindrical, smooth- and thick-walled, 6–13.5 × 3–5.5 μm. Secondary ramoconidia oblong, pale brown, 0–1 septate, 2–4 distal hila, 5.5–9 × 2.5–5 μm. Conidia numerous, catenate, forming short branched chains, aseptate, olive to brown, smooth- and thin- walled, ellipsoid-ovoid, obovoid, 2–7.5 × 2–4 μm. Intercalary conidia limoniform, oval to ellipsoid, with hila protuberant, 3.5–7.5 × 2.5–4 μm. Terminal conidia globose to ellipsoid, sometimes hila evident, 2–4 × 2–3.5 μm. Microcyclic conidiogenesis absent. In vitro on SNA: Mycelium abundant, submerged, overgrowing whole culture dishes, hyphae straight to slightly sinuous, septate and branched, olive to brown, and thick-walled, 2–3.5 µm wide. Conidiophores erect, occasionally branched, brown, thick-walled, 33–118 × 2.5–4 µm. Ramoconidia olive to brown, narrowly ellipsoid to cylindrical-oblong, subcylindrical, septate or aseptate, smooth- and thick-walled, 6–17.5 (–20) × 2–5 µm. Conidia in simple and branched acropetal chains, light olive, aseptate, smooth- and thin-walled, ellipsoidovoid, obovoid, fusiform, 3–6.5 × 2–4 µm. Intercalary conidia oval to ellipsoid, with hila protuberant, 3.5–6.5 × 2.5–4 μm. Terminal conidia globose to ellipsoid, 2.5–4 × 2–4 μm.

**Figure 4.**
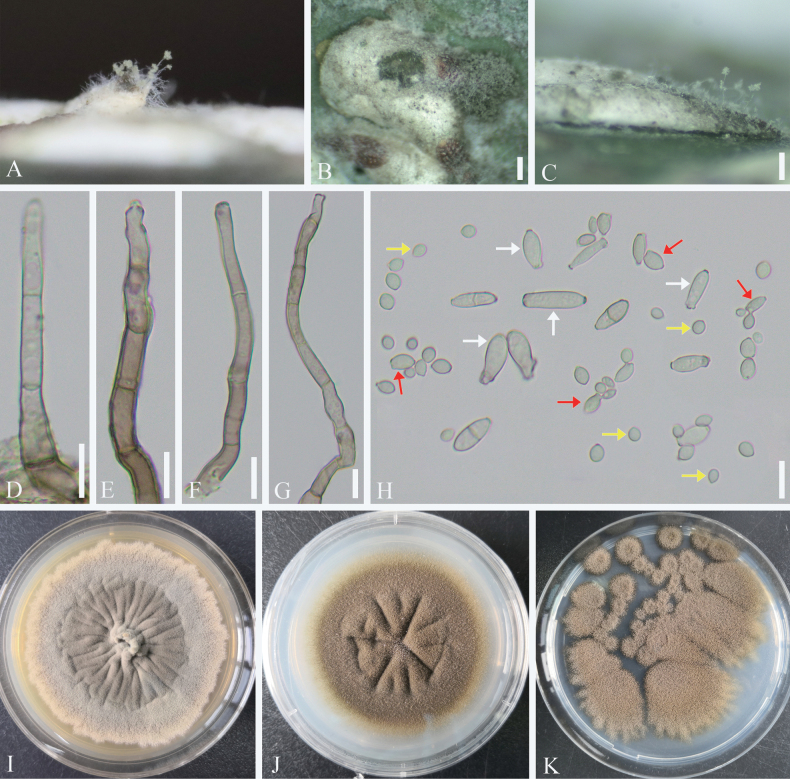
*Cladosporium kuwanaspidis*. A–C. Symptoms and appearance of colonies observed on *Kuwanaspis howardi*. D–G. Conidiophores. H. Ramoconidia (white arrows), secondary ramoconidia (red arrows), and terminal conidia (yellow arrows). I–K. Colonies on MEA, PDA, and SNA for 7 days. Scale bars: 200 µm (B, C); 10 µm (D–H).

##### Culture characteristics.

Conidia of all types can germinate within 12 h in sterile distilled water, with germination tubes forming from any part of the conidial body. Colonies on MEA attaining 40–50 mm diameter, after 1 week at 25 °C, greyish-green to greyish toward regular margins, velvety, sporulation profuse, radially furrowed, aerial mycelium abundant, reverse dark green. Colonies on PDA attaining 35–40 mm diameter, after 1 week at 25 °C, grey-olivaceous to iron grey, pale greenish grey toward regular margins, reverse dark green. Colonies on SNA attaining 20–30 mm diameter, after 1 week at 25 °C, smoke-grey to olivaceous-grey, aerial mycelium loose, reverse olivaceous to brown.

##### Host.

*Kuwanaspis howardi* (*Diaspididae*).

##### Habitat.

Subtropical evergreen broad-leaved forests, particularly bamboo forests, are common habitats. *Kuwanaspis howardi* often causes damage in the lower canopy of bamboo forests. Occasionally, *Cladosporium kuwanaspidis* can be observed on bamboo culms. Scale insects are more easily found in shaded and sheltered areas.

##### Distribution.

China, Sichuan Province, Meishan City.

##### Material examined.

**CHINA** • Sichuan Province, Meishan City, Hongya County. Infected scale insects (*Kuwanaspis howardi*) were found on the culms of bamboo (*Pleioblastus amarus*), 29°41.88'N, 103°14.04'E, alt. 540 m, 9 Mar. 2021, C.L. Yang, YCL202103004 (living culture SICAUCC 25-0063); • *ibid.* YCL202103004-1 (SICAU 25-0083, living culture SICAUCC 25-0064).

##### Notes.

The ITS base-pair comparison between *Cladosporium kuwanaspidis* (SICAUCC 25-0063) and the phylogenetically affiliated ex-type culture of *C. perangustum* (CBS 125996) shows no nucleotide differences. However, nucleotide differences in the act and *tef*1-α regions are 1.86% (4 bp, 0 gap) and 4.01% (15 bp, 2 gaps), respectively. Morphologically, *C. perangustum* has larger ramoconidia compared to those observed in *C. kuwanaspidis* on both the host and SNA medium (25–45 µm vs. 6–13.5 µm and 6–20 µm, respectively). In addition, compared to the length of intercalary conidia (4–19 µm) observed in *C. perangustum* (CBS 125996), our isolates exhibit shorter intercalary conidia on both the host and PDA medium (3.5–7.5 µm and 3.5–6.5 µm, respectively) (Bensch et al. 2010).

#### ﻿ Cladosporium guizhouense

Taxon classificationAnimaliaCladosporialesCladosporiaceae

S.Y. Wang, Yong Wang bis & Y. Li, MycoKeys 91: 151–168 (2022) sp. nov.

ED7038F0-BC36-58B0-BC5E-62217B81C6E3

MB 842407

Fig. 5

##### Description.

Parasitic on aphids from *Telosma cordata* (Burm. f.) Merr. (*Apocynaceae*). **Sexual morph**: Not observed. **Asexual morph**: Hyphomycetous.

Mycelium superficial and immersed, with abundant sporulation on the surface of aphids. Conidiophores erect, fasciculate, macronematous, cylindrical, subnodulose or nodulose, geniculate, septate, branched, pale brown, slightly roughened to verruculose, thick-walled, and pronounced loci, 111–367 × 3.5–5 μm. Conidiogenous cells integrated, terminal or intercalary, cylindrical, cylindrical-oblong, sometimes geniculate, 20–65 × 3–6 μm, conidiogenous loci at the apex (2–5) or in lateral shoulders (0–4). Ramoconidia olive-green, 0–4-septate, ellipsoidal to subcylindrical, smooth- and thick-walled, 10–26 × 3.5–7.5 μm. Secondary ramoconidia pale brown, oblong, oblong-ellipsoid, 0–1septate, 1–4 distal hila, 8.5–13.5 × 3.5–7.5 μm. Conidia numerous, catenate, forming short branched chains, aseptate, olive to light olive, smooth- and thin-walled, variable in size and shape, ellipsoid-ovoid, obovoid, and fusiform, 4–10 × 3–6.5 μm. Intercalary conidia aseptate, olive to light olive, ellipsoid-ovoid, fusiform, 6–10 × 4–6.5 μm. Terminal conidia aseptate, olive to light olive, obovoid, 4–6.5 × 3–5.5 μm. Microcyclic conidiogenesis absent. In vitro on SNA: Mycelium abundant, submerged, overgrowing whole culture dishes, hyphae straight to slightly sinuous, septate and branched, light olive-green to olive-green, and thick-walled, 1.5–4.5 µm wide. Conidiophores erect, branched, light olive-green, thick-walled, 43–163 × 2.5–4 µm. Ramoconidia pale olivegreen, narrowly ellipsoid to cylindrical-oblong, subcylindrical, septate or aseptate, smooth- and thick-walled, 6–27 × 3–5 µm. Conidia in simple and branched acropetal chains, light olive, aseptate, smooth- and thin-walled, variable in size and shape, ellipsoid-ovoid, obovoid, fusiform, 3–8 × 2.5–4 µm. Intercalary conidia light olive, aseptate, smooth- and thin-walled, mostly ellipsoid, 3.5–8 × 2.5–4 µm. Terminal conidia light olive, aseptate, smooth- and thin-walled, mostly ovoid, 3–4.5 × 2.5–3.5 µm.

**Figure 5.**
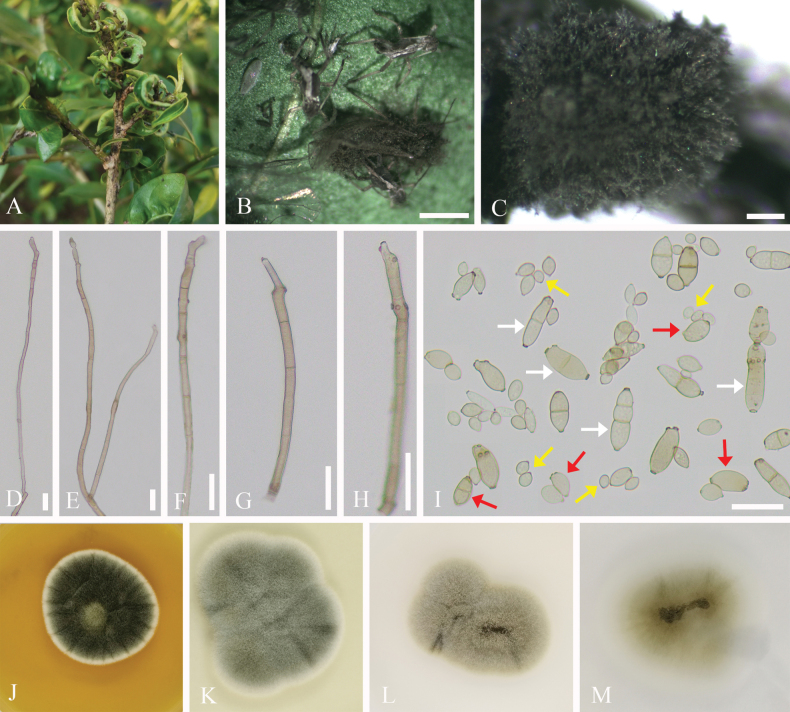
*Cladosporium guizhouense*. A. Symptoms observed on host. B, C. Appearance of colonies on aphids. D–H. Conidiophores. I. Ramoconidia (white arrows), secondary ramoconidia (red arrows), and terminal conidia (yellow arrows). J–M. Colonies on MEA, PDA, OA, and SNA for 7 days. Scale bars: 1000 µm (B); 200 µm (C); 20 µm (D–I).

##### Culture characteristics.

All conidia can germinate within 12 h in sterile distilled water, with tubes forming from any part. Colonies on MEA attaining 35–45 mm diameter, after 1 week at 25 °C, dark green, white to greyish toward regular margins, velvety, sporulation profuse, radially furrowed, aerial mycelium abundant, reverse dark green. Colonies on PDA attaining 30–40 mm diameter, after 1 week at 25 °C, greyishgreen, greyish toward irregular margins, velvety, sporulation profuse, radially furrowed, aerial mycelium abundant, reverse dark green. Colonies on OA attaining 30–40 mm diameter, after 1 week at 25 °C, greyish olivaceous, white toward irregular margins, sporulation profuse, reverse olivaceous. Colonies on SNA attaining 25–30 mm diameter, after 1 week at 25 °C, olivaceous, flat, white toward regular and wide margins, aerial mycelium loose, reverse olivaceous.

##### Host.

Aphids (*Aphididae*), leaves of plants, and uredinia of *Hemileia vastatrix* (*Pucciniaceae*).

##### Habitat.

In tropical and subtropical regions, it can infect insects, act saprophytically on plant tissues, and function as a fungicolous fungus on the uredinia of rust. During the middle and late stages of aphid damage, numerous aphids die on the underside of plant leaves, becoming enveloped in mycelial tissue.

##### Distribution.

China, Sichuan Province, Guangan City. China, Guizhou Province, Guiyang City. Ethiopia, Oromia Region, Illubabor Gore. Brazil, Minas Gerais, Viçosa.

##### Material examined.

**CHINA** • Sichuan Province, Guangan City, Yuechi County. Infected aphids were found on the underside of the leaves of *Telosma cordata*, 30°44.18"N, 106°30.80"E, alt. 480 m, 31 Jan. 2020, X.L. Xu, XXL202001003 (SICAU 25-0076, living culture SICAUCC 25-0057); • *ibid.* XXL202001003-1 (SICAU 25-0077, living culture SICAUCC 25-0058).

##### Notes.

Phylogenetically, our collections grouped with isolates of *Cladosporium guizhouense* within the *C. cladosporioides* complex, showing strong statistical support (92% MLBS, 0.97 BIPP) (Fig. 1). A total of 6 bp nucleotide differences were observed between our isolate SICAUCC 25-0057 and the ex-type culture of *C. guizhouense* (GUCC 401.7), with 0 bp in ITS, 2 bp in *act*, and 4 bp in *tef*1-α (Wang et al. 2022b). Morphologically, our isolate is fully consistent with *C. guizhouense*, showing no noticeable differences (Wang et al. 2022b; Pereira et al. 2024).

#### ﻿ Moelleriella eucalypti

Taxon classificationAnimaliaHypocrealesClavicipitaceae

X.L. Xu, Feng Liu & C.L. Yang sp. nov.

D19B1D61-701B-506C-BBEF-3202B64D2F12

MB 858370

Fig. 6

##### Etymology.

In reference to the generic name of host plants.

##### Diagnosis.

Similar to *Moelleriella sinensis* in having somewhat similar stromata, *M. eucalypti* differs by having longer and wider paraphyses, shorter and wider conidiogenous cells, and wider conidia.

##### Type.

**CHINA** • Sichuan Province, Dazhou City, Heibaoshan National Forest Park. Infected scale insects were found on the underside of leaves of *Eucalyptus* sp. (*Myrtaceae*), 31°55.30'N, 107°47.75'E, alt. 690 m, 21 Jul. 2022, Feng Liu, LF202207001A (SICAU 25-0072 – holotype preserved in the Herbarium of Sichuan Agricultural University).

##### Description.

Parasitic on scale insects found on eucalyptus leaves. **Sexual morph**: Not observed. **Asexual morph**: Coelomycetous. Stromata yellow to dark orange in fresh specimens, pale yellow to white in old, entirely covering the insect hosts, thickened pulvinate, umbonate to hemispheric, cottony, sessile, globose to subglobose, tubercules on the surface, 1.5–2.5 mm in diameter, 1–2 mm in high. Conidiomata orifice scattered or circularly arranged, oval or elongate flask shaped, narrow orifices, 120–460 × 80–155 μm. Phialides hyaline, stick-shaped to cylindrical, 10–18 × 1–2.5 μm. Conidia hyaline, fusiform, yellow conidial masses, 8–12 × 1.5–3 μm. Paraphyses present, hyaline, filiform, 90–180 × 1.0–1.6 μm.

**Figure 6.**
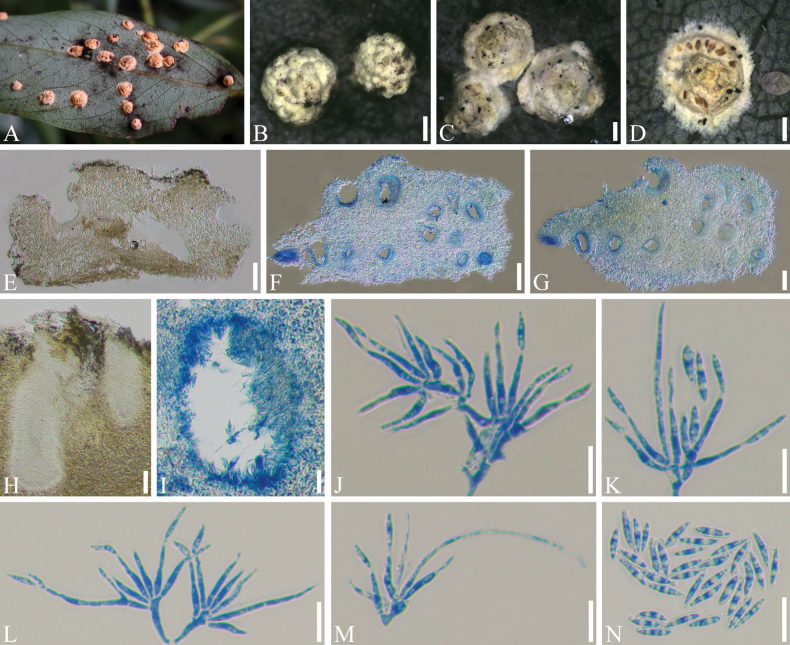
*Moelleriella eucalypti*. A. Fungus-infected scale insect on the underside of leaves. B–D. Stromata containing conidiomata with conidial masses. E–I. Section of stroma showing conidioma. J–M. Phialides and conidia at the tips with paraphyses. N. Conidia. Scale bars: 500 mm (B–D); 200 μm (E, F); 50 μm (H, I); 10 μm (J, K).

##### Culture characteristics.

No germination was observed due to the specimens being dried and the culture not being obtained.

##### Host.

Scale insects (*Coccidae*).

##### Habitat.

Subtropical monsoon evergreen broad-leaf forest. Scale insects were infected on the underside of eucalyptus leaves.

##### Distribution.

China, Sichuan Province, Dazhou City.

##### Material examined.

**CHINA** • Sichuan Province, Dazhou City, Heibaoshan National Forest Park. Infected scale insects were found on the underside of leaves of *Eucalyptus* sp., 31°55.30'N, 107°47.75'E, alt. 690 m, 21 Jul. 2022, Feng Liu, LF202207001A (SICAU 25-0072); • *ibid.* LF202207001B (SICAU 25-0073); • *ibid.* LF202207001C (SICAU 25-0074); • *ibid.* LF202207001D (SICAU 25-0075).

##### Notes.

Phylogenetically, our collections are closely related to *Moelleriella sinensis*, showing few sequence differences with the type strain CGMCC3.18911, specifically 1 bp in LSU (0.12%, 0 gap) and 8 bp in *rpb*1 (1.10%, 0 gap), while the *tef*1-α sequence is invalid. *Moelleriella sinensis* was first described on whitefly nymphs (Hemiptera) and later found on scale insects (*Coccidae*) in Thailand (Chen et al. 2020; Khonsanit et al. 2021). The described specimens of *M. sinensis* are primarily characterized by flat to umbonate, pale yellow pulvinate stromata and fusiform conidia. However, our collections differ from *M. sinensis* in having pulvinate stromata that are yellow to dark orange when fresh, pale yellow to white when old, and thicker in appearance. Additionally, they have longer and wider paraphyses (90–180 × 1.0–1.6 μm vs. 43.2–68.9 × 0.6–0.8 μm), shorter and wider conidiogenous cells (10–18 × 1–2.5 μm vs. up to 30 μm × 0.8–1.3 μm), and wider conidia (1.5–3 μm vs. 1.3–1.8 μm). Hence, we introduce *M. eucalypti* as a new species, based on the distinct morphological differences observed.

#### ﻿ Moelleriella boehmeriae

Taxon classificationAnimaliaHypocrealesClavicipitaceae

X.L. Xu & C.L. Yang sp. nov.

D057A820-33B7-5F5C-A339-0D4170E17819

MB 858372

Fig. 7

##### Etymology.

In reference to the generic name of host plants.

##### Diagnosis.

Similar to *Moelleriella jinuoana* in having yellow to orange, globose stromata with a narrow hypothallus, *M. boehmeriae* differs by its slower growth on PDA, unique colony characteristics, and wider conidia.

##### Type.

**CHINA** • Sichuan Province, Leshan City, Muchuan County. Infected scale insects were found on the stems of *Boehmeria spicata* (Thunb.) Thunb., 28°47.91'N, 103°55.63'E, alt. 900 m, 12 Mar. 2021, C.L. Yang, YCL202103003 (SICAU 25-0080 – holotype preserved in the Herbarium of Sichuan Agricultural University; living culture SICAUCC 25-0061 – ex-holotype stored in the Culture Collection in Sichuan Agricultural University).

##### Description.

Parasitic on scale insect from *Boehmeria spicata* (*Urticaceae*). **Sexual morph**: Not observed. **Asexual morph**: Coelomycetous. Stromata yellow to orange in fresh specimens, entirely covering the insect hosts, thickened pulvinate, globose, tubercules on the surface, closely aggregated, some with narrow hypothallus, 0.8–2.5 mm diameter. Hyphae of stromata form compact textura epidermoidea. Conidiomata simple depressions of surface, round or irregular shape, no mature conidiomata observed.

**Figure 7.**
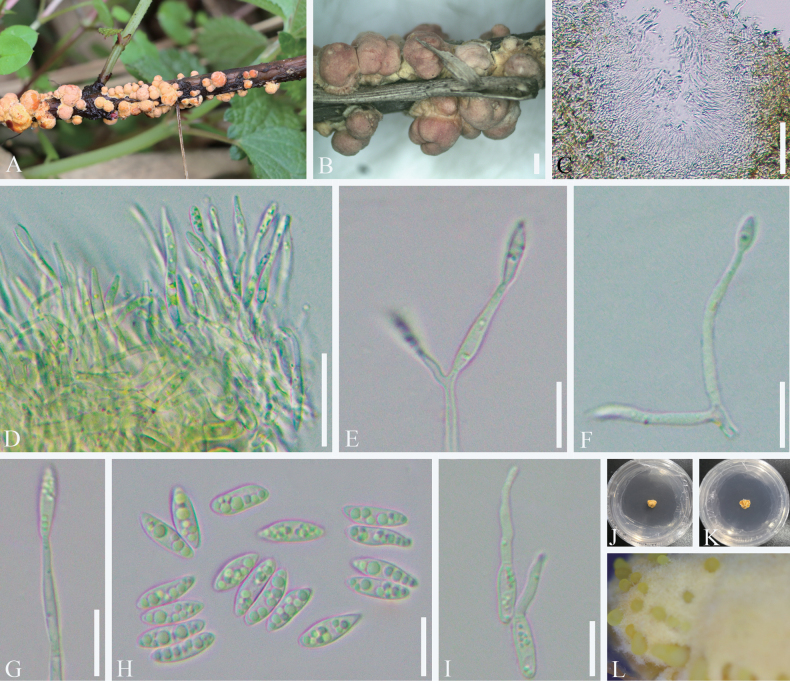
*Moelleriella boehmeriae*. A, B. Immature stromata over scale insects. C. Conidioma formed in PDA. D. Phialides with conidia at the tips. E–G. Phialides with developing conidia. H. Conidia. I. Germinating conidia. J, K. Colonies obverse and reverse on PDA at 25 °C after 3 weeks. L. Conidial mass on PDA. Scale bars: 1000 µm (B); 50 µm (C); 20 µm (D); 10 µm (E–I).

##### Culture characteristics.

The conidia germinate within 12 h in sterile distilled water, with germ tubes extending from both ends of the conidia. Colonies on PDA slowgrowing, attaining a diameter of 5–7 mm in 28 days at 25 °C. Colonies compact pulvinate, surface velutinous, pale yellow to yellow. Conidial masses usually abundant, yellow. Reverse of colony pale orange. Conidiomata U-shaped, subcircular to circular. Conidia unicellular, hyaline, smooth, fusoid with rounded ends, 9.5–12.5 × 3–4 µm. No paraphyses were observed.

##### Host.

Scale insects (*Coccidae*).

##### Habitat.

Subtropical monsoon evergreen broad-leaf forests serve as the habitat for *Moelleriella boehmeriae*, which can infect scale insects found on the stems of *Boehmeria spicata*. Infections are typically observed at lower heights, close to the ground.

##### Distribution.

China, Sichuan Province, Leshan City.

##### Material examined.

**CHINA** • Sichuan Province, Leshan City, Muchuan County. Infected scale insects were found on the stems of *Boehmeria spicata*, 28°47.91'N, 103°55.63'E, alt. 900 m, 12 Mar. 2021, C.L. Yang, YCL202103003 (SICAU 25-0080, living culture SICAUCC 25-0061); • *ibid.* YCL202103003-1 (SICAU 25-0081, living culture SICAUCC 25-0062).

##### Notes.

Phylogenetic analysis revealed that *Moelleriella boehmeriae* clusters within the Globose clade and is closely related to *M. jinuoana* (Fig. 2), sharing characteristics such as yellow to orange, globose stromata with a narrow hypothallus (Wang et al. 2024). Although detailed morphological comparisons were limited due to the absence of mature conidiomata in our specimen, *M. boehmeriae* differs by its slower growth on PDA and distinct colony characteristics. The colonies of *M. boehmeriae* are yellow on the front and light orange on the reverse, whereas those of *M. jinuoana* are pale orange to orange on the front and brownish on the reverse. Additionally, *M. boehmeriae* has wider conidia (3–4 µm) observed in culture compared to *M. jinuoana*, which has conidia measuring 2.1–2.9 µm on the substrate. In addition, *M. boehmeriae* differs from the type strain *M. jinuoana* (YHH MJBP2309031) by 16 bp (1.76%, 0 gap) in the *tef*1α region, 2 bp (0.28%, 0 gap) in the *rpb*1 region, and 4 bp (0.47%, 2 gaps) in the LSU region, respectively.

#### ﻿ Moelleriella cinnamomum

Taxon classificationAnimaliaHypocrealesClavicipitaceae

X.L. Xu & C.L. Yang sp. nov.

EA4791CE-91B4-574B-98EC-EE4590D1454F

MB 858373

Fig. 8

##### Etymology.

In reference to the generic name of host plants.

##### Diagnosis.

Similar to *Moelleriella simaoensis*, *M. puerensis*, *M. raciborskii*, and *M. citrus*, *M. cinnamomum* differs by having red-orange, thickened pulvinate stromata, longer conidia and paraphyses, and the absence of a hypothallus surrounding the stroma.

##### Type.

**CHINA** • Sichuan Province, Chengdu City, Chongzhou County. Infected scale insects were found on the underside of leaves of *Cinnamomum cassia* (L.) D. Don., 30°48.82'N, 103°31.52'E, alt. 851 m, 1 May 2021, C.L. Yang, YCL202105001 (SICAU 25-0086 – holotype preserved in the Herbarium of Sichuan Agricultural University; living culture SICAUCC 25-0067 – ex-holotype stored in the Culture Collection in Sichuan Agricultural University).

##### Description.

Parasitic on scale insects found on the underside of leaves of *Cinnamomum cassia* (*Lauraceae*). **Sexual morph**: Not observed. **Asexual morph**: Coelomycetous. Stromata red-orange in fresh specimens, mostly solitary, sometimes gregarious, globose to subglobose, thickened pulvinate up to 1 mm, 0.9–2.8 mm in diameter. Conidiomata aggregated in the center of the stroma and widely open, simple depressions of the surface without distinct rims. In section, conidioma flask-shaped or irregular, shallow. Conidial masses orange. Conidia hyaline, smooth, one-celled, fusoid, with acute ends, 13–16.5 × 2–3 µm. Paraphyses present, linear, filiform, up to 102 µm long.

**Figure 8.**
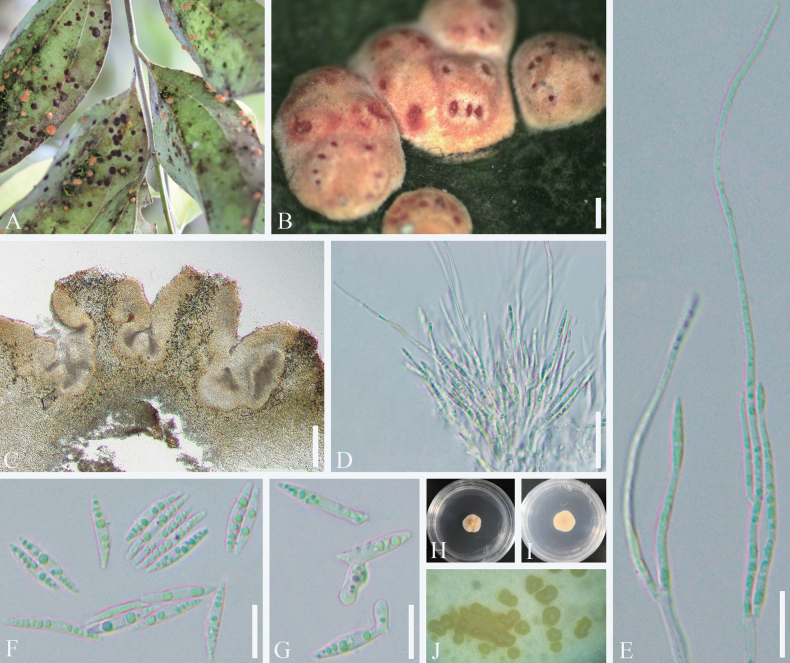
*Moelleriella cinnamomum*. A, B. Stromata over scale insects on the underside of leaves. C. Section of stroma showing conidioma. D. Phialides and conidia at the tips with paraphyses. E. Paraphyses with phialides bearing developing conidia. F. Conidia. G. Germinating conidia. H, I. Colonies obverse and reverse on PDA at 25 °C after 3 weeks. J. Conidial mass on PDA. Scale bars: 1000 µm (B); 500 µm (C); 20 µm (D); 10 µm (E–G).

##### Culture characteristics.

Conidia germinate in sterile water within 12 h, with germination tubes typically growing laterally from both ends of the spore and occasionally from the middle. Colonies on PDA at 25 °C attaining 15 mm diameter in 20 days. Colonies pale yellow and formed compact pulvinate with abundant slimy masses. Conidial masses light yellow. Colonies reverse dark yellow with pale yellow margins. Conidia 9–15 × 1.8–2.8 µm. Paraphyses rare.

##### Host.

Scale insects (*Coccidae*).

##### Habitat.

Subtropical monsoon evergreen broad-leaf forests serve as the habitat for *Moelleriella cinnamomum*, which can infect scale insects found on the underside of *Cinnamomum cassia* leaves. Infections are typically observed throughout the trees, particularly in the lower canopy.

##### Distribution.

China, Sichuan Province, Chengdu City.

##### Material examined.

**CHINA** • Sichuan Province, Chengdu City, Chongzhou County. Infected scale insects were found on the underside of leaves of *Cinnamomum cassia*, 30°48'49.57"N, 103°31'31.19"E, alt. 851 m, 1 May 2021, C.L. Yang, YCL202105001 (SICAU 25-0086, living culture SICAUCC 25-0067); • *ibid.* YCL202105002 (SICAU 25-0087, living culture SICAUCC 25-0068).

##### Notes.

Our collections were nested in the *Moelleriella* clade related to *M. citrus*, *M. raciborskii*, *M. simaoensis*, *M. hainanensis*, *M. puerensis*, and *M. pseudothanathonensis* (Fig. 2). Morphologically, *M. cinnamomum* is distinct from the phylogenetically related six species, with red-orange and thickened pulvinate, while the other species are flattened and more pale in color (Liu et al. 2006; Chaverri et al. 2008; Wang et al. 2022c, 2025; Yang et al. 2023a). *Moelleriella raciborskii*, *M. simaoensis*, and *M. puerensis* can co-occur in the same stromata with both sexual and asexual morphs, whereas only the asexual morph of *M. cinnamomum* has been observed in stromatal tissue. *Moelleriella hainanensis* differs from *M. cinnamomum* by having larger stromata (3–4 mm vs. 0.9–2.8 mm), and along with *M. raciborskii* and *M. citrus*, it also differs by exhibiting a light-colored hypothallus surrounding the stroma. Conidia in *M. cinnamomum* (13–16.5 µm) are longer than those of *M. simaoensis* (8.8–14 µm), *M. puerensis* (9.7–13.4 µm), *M. pseudothanathonensis* (10–12.5 µm), and *M. raciborskii* (11–14 µm). Moreover, *M. cinnamomum* has longer paraphyses (up to 102 µm) than *M. citrus* (85–100 µm), *M. simaoensis* (up to 95 µm) and *M. raciborskii* (40–70 µm).

#### ﻿ Moelleriella citrus

Taxon classificationAnimaliaHypocrealesClavicipitaceae

X.L. Xu & C.L. Yang sp. nov.

8D509E83-9215-5AB0-B171-097096DB3BC4

MB 858374

Fig. 9

##### Etymology.

In reference to the generic name of host plants.

##### Diagnosis.

Similar to *Moelleriella raciborskii* in having pale yellow, thin, pulvinate stromata, *M. citrus* differs by its longer paraphyses and wider conidia.

##### Type.

**CHINA** • Sichuan Province, Chengdu City, Wenjiang District. Infected scale insects were found on the underside of leaves of *Citrus* sp., 30°42.40'N, 103°51.05'E, alt. 590 m, 10 Oct. 2020, X.L. Xu, XXL202010001 (SICAU 25-0078 – holotype preserved in the Herbarium of Sichuan Agricultural University; living culture SICAUCC 25-0059 – ex-holotype stored in the Culture Collection in Sichuan Agricultural University).

##### Description.

Parasitic on scale insect from citrus leaves (*Rutaceae*). **Sexual morph**: Not observed. **Asexual morph**: Coelomycetous. Stromata orange in fresh specimens, globose to subglobose, flattened pulvinate, 1.8–3.0 mm in diameter, surrounded by a hyaline hypothallus up to 1.6 mm wide and covered with confluent conidial masses of orange-yellow. Conidiomata simple and aggregated in the center of the stroma, and widely opened. Conidial masses orange. Conidia hyaline, smooth, one-celled, fusoid, with acute ends, produced in copious slime, 12–18.5 × 2.3–3.2 µm. Paraphyses present, arising from the hymenium of the conidioma, filiform, tapering at the apices, up to 85–100 µm long.

**Figure 9.**
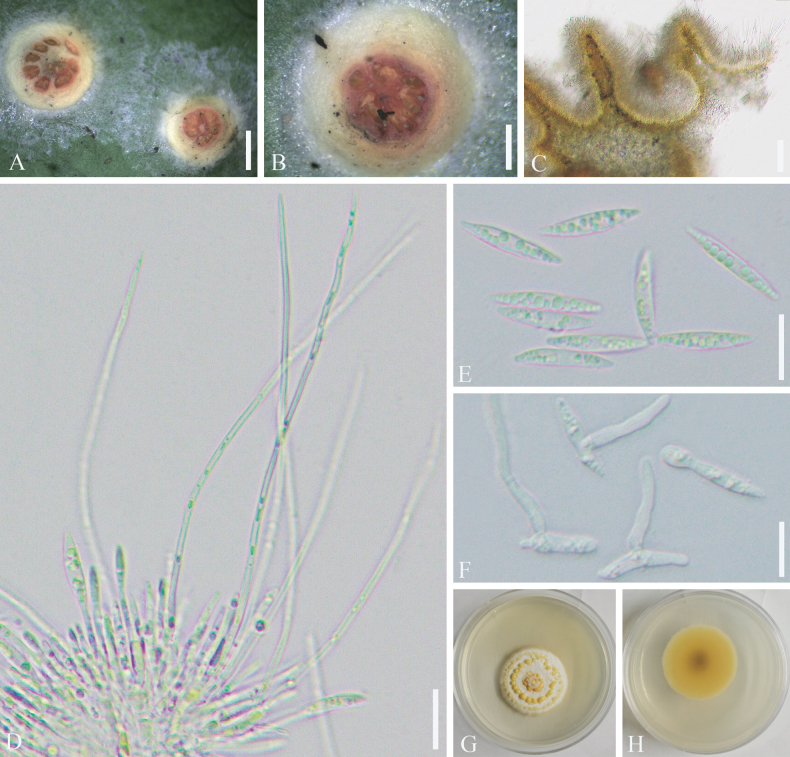
*Moelleriella citrus*. A, B. Fungus-infected scale insect on the underside of leaves. C. Section of stroma showing conidioma. D. Phialides and conidia at the tips with paraphyses. E. Conidia. F. Germinating conidia. G, H. Colonies obverse and reverse on PDA at 25 °C after 3 weeks. Scale bars: 1000 µm (A); 500 µm (B); 50 µm (C); 10 µm (D–F).

##### Culture characteristics.

Conidia germinate in sterile water within 12 h, with germination tubes typically growing laterally from both ends of the spore. Colonies on PDA at 25 °C attaining 25 mm diameter in 20 days. Colonies pale yellow and formed compact pulvinate with abundant slimy masses. Conidial masses light yellow, the colony reverse dark yellow, and the margins pale yellow. Conidia hyaline, smooth, onecelled, fusoid, 12–15.5 × 2.0–2.8 µm. Paraphyses rare.

##### Host.

Scale insects (*Coccidae*).

##### Habitat.

Subtropical monsoon evergreen broad-leaf forests serve as the habitat for *Moelleriella citrus*, which infects scale insects found on the underside of *Citrus* sp. leaves. Infections are typically observed throughout the trees.

##### Distribution.

China, Sichuan Province, Chengdu City.

##### Material examined.

**CHINA** • Sichuan Province, Chengdu City, Wenjiang District. Infected scale insects were found on the underside of leaves of *Citrus* sp., 30°42.40'N, 103°51.05'E, alt. 590 m, 10 Oct. 2020, X.L. Xu, XXL202010001 (SICAU 25-0078, living culture SICAUCC 25-0059); • *ibid.* XXL202010001-1 (SICAU 25-0079, living culture SICAUCC 25-0060).

##### Notes.

*Moelleriella raciborskii* is accepted as the teleomorph of *Aschersonia placenta*, which was previously linked to *Hypocrella raciborskii* (Chaverri et al. 2008). Morphologically, *M. citrus* is closely related to *M. raciborskii*, sharing characteristics such as a pale yellow, thin, pulvinate stroma, ovoid or subglobose tubercle processes developing on the periphery of the stroma, and orange ostioles. However, the paraphyses show differences in length, with our collection measuring 85–100 µm compared to 40–70 µm in the previous species, and our collection has larger conidia (12–18.5 × 2.3–3.2 µm vs. 11–14 × 1.5–2 µm) (Liu et al. 2006).

#### ﻿ Podonectria multiarmata

Taxon classificationAnimaliaPleosporalesPodonectriaceae

X.L. Xu & C.L. Yang sp. nov.

5B0521AE-FEA9-599F-B7AC-09D233734A3A

MB 85837

Fig. 10

##### Etymology.

Refers to the species having conidia bearing multiple arm-like extensions.

##### Diagnosis.

Similar to species of *Podonectria*, *P. multiarmata* differs notably in sporulation and conidial morphology, particularly in the extension patterns, size, and septation of the conidial arms.

##### Type.

**CHINA** • Sichuan Province, Meishan City, Hongya County. Infected scale insects (*Kuwanaspis howardi*) were found on the culms of bamboo (*Pleioblastus amarus*), 29°41.88'N, 103°14.04'E, alt. 540 m, 13 Mar. 2021, C.L. Yang, YCL202103005 (SICAU 25-0084 – holotype preserved in the Herbarium of Sichuan Agricultural University; living culture SICAUCC 25-0065 – ex-holotype stored in the Culture Collection in Sichuan Agricultural University).

##### Description.

Habitat associated with scale insects *Kuwanaspis howardi* on *Pleioblastus amarus* (*Poaceae*). ***Sexual morph***: Not observed. ***Asexual morph***: *Hyphomycetes*. Colonies surround the scale insects, effuse, white, and diffuse outward to produce thin layers of hyphae. Mycelia branched, septate, 1–3 µm wide. Conidiophores inconspicuous, mononematous, short, straight or slightly curved, mostly reduced to conidiogenous cells. Conidiogenous cells subglobose to globose, acrogenous, determinate hyaline, with obviously conidiogenous loci, 4–6 × 4–7 µm. Conidia usually with two and three arms, V-shaped, Y-shaped or T-shaped, occasionally four arms, smooth, hyaline, each arm varies in length and sharply divergent, slightly constricted at the septa, straight or curved, and tapering toward the apical cell, 1–4septate for each arm, 20–40 (–50) µm long, 3.5–6 wide at the base, 1.5–3 wide near the apex. All arms arise from a bigger basal cell, measuring 6–8 µm wide.

**Figure 10.**
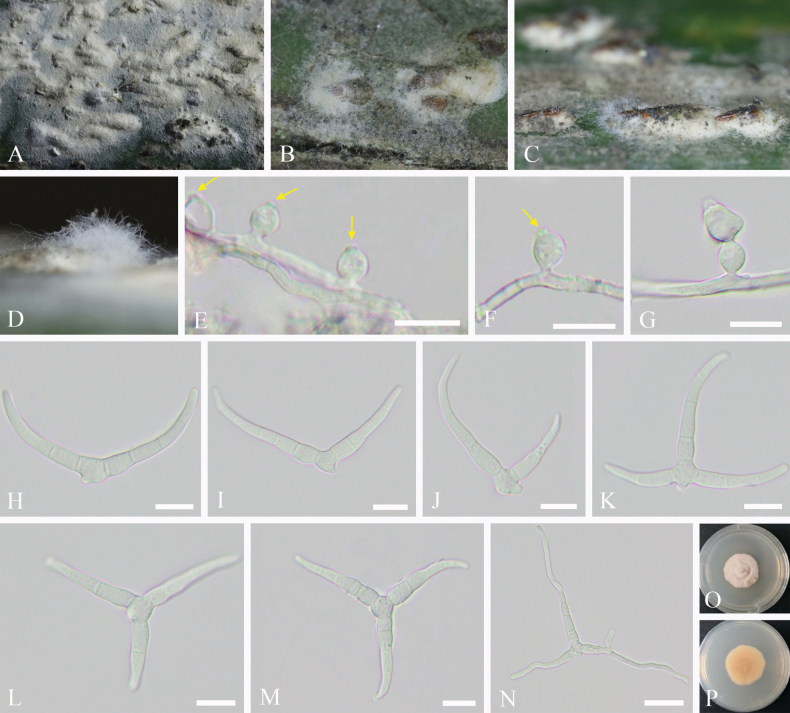
*Podonectria multiarmata*. A–D. Views of colonies of scale insects. E, F. Conidiogenous cells with loci at the apex (yellow arrows). G. Conidiogenous cell with developing conidium. H–M. Conidia. N. Germinating conidium. O, P. Upper and reverse views of cultures on PDA after 3 weeks. Scale bars: 10 μm (E–M); 20 μm (N).

##### Culture characteristics.

Conidia germinate on PDA within 12 h, with the germination tube usually growing from the tip of the arm. Colonies grow slowly on PDA, reaching 2.5 cm in diameter after 20 days at 25 °C, flat, circular, white, gradually becoming light fleshy pink, and the back of colonies yellow.

##### Host.

*Kuwanaspis howardi* (*Coccidae*).

##### Habitat.

Subtropical monsoon evergreen broad-leaf forests serve as the habitat for *Podonectria multiarmata*, which infects scale insects found on the culms of bamboo. Infections are typically observed in moist, shaded environments.

##### Distribution.

China, Sichuan Province, Meishan City.

##### Material examined.

**CHINA** • Sichuan Province, Meishan City, Hongya County. Infected scale insects (*Kuwanaspis howardi*) were found on the culms of *Pleioblastus amarus*, 29°41.88'N, 103°14.04'E, alt. 540 m, 15 Mar. 2021, C.L. Yang, YCL202103005 (SICAU 25-0084, living culture SICAUCC 25-0065); • *ibid.* YCL202103005-1 (SICAU 25-0085, living culture SICAUCC 25-0066).

##### Notes.

Our collections clustered phylogenetically with species of *Podonectria* based on combined ITS, LSU, SSU, *rpb*2, and *tef*1-α sequence data (Fig. 3) (100% MLBS, 1.00 BIPP). Nucleotide BLAST sequence showed distinguishing identities with those related species *P. sichuanensis* (SICAU 16-0003), *P. coccicola* (pAak), *P. novae-zelandiae* (SICAUCC 21-0004), and *P. kuwanaspidis* (SICAUCC 21-0002) in ITS (90.38%, 88.45%, 89.89%, 88.26%), LSU (96.77%, 98.26%, 97.92%, 98.62%), SSU (99.62%, -, 99.81%, 100%), *tef*1-a (95.34%, -, 94.35%, 95.34%), and *rpb*2 (93.20%, -, 90.74%, 90.39%), respectively. *Podonectria multiarmata* resembles other *Podonectria* species in its asexual state, producing conidia with arms that are joined at a basal cell (Xu et al. 2021a). However, it differs in the length and divergence angle of each arm, and it also produces T-type and Y-type conidia with radiating divergent arms.

## ﻿Discussion

*Hypocreales (Ascomycota)* and *Entomophthoromycotina (Zoopagomycota)* harbor the most common insect-pathogenic fungi (Hong et al. 2024), while insects in the *Coccidae* and *Aleyrodidae* exhibit the greatest documented diversity of fungal pathogens (Humber 2008). In this study, we identified seven species of entomopathogenic fungi associated with scale insects and aphids, belonging to *Cladosporium*, *Moelleriella*, and *Podonectria*, based on morphology and phylogeny. These findings provide valuable insights to support the further development and application of effective biological control strategies against pests.

*Cladosporium* is a genus in the family *Cladosporiaceae* of *Dothideomycetes* (Abdollahzadeh et al. 2020), isolated from a wide range of substrates and known as saprobes, endophytes, plant pathogens, and hyperparasites of other fungi and etiologic agents in vertebrate hosts, including humans (El-Morsy 2000; Abdel-Baky and Abdel-Salam 2003; Heuchert et al. 2005; Levetin and Dorsey 2006; Bensch et al. 2012; Sandoval-Denis et al. 2016; Prasannath et al. 2021a, 2021b; Xu et al. 2022; Pereira et al. 2024). The classification of *Cladosporium* is problematic due to the infrequency of the teleomorphic stage and the similarity in conidial structures. Mycologists have resolved the complexes of *C. cladosporioides*, *C. herbarum*, and *C. sphaerospermum* with the help of biomolecular tools. The occurrence of *Cladosporium* species within these three complexes, in association with insects, was summarized from the available literature by Nicoletti et al. (2024). In that list, about 46% of the entries were not identified due to the challenging classification of *Cladosporium*, and the insect-associated species were predominantly linked to Hemiptera (28.2%) and *Coleoptera* (27.6%). *Cladosporium guizhouense* seems to have a geographical distribution with a broad host range, based on records from leaves of *Eucommia ulmoides* (*Eucommiaceae*) (Wang et al. 2022b) and *Citrus reticulata* (*Rutaceae*) (Yang et al. 2023b). In addition, three isolates of *C. guizhouense* were obtained from uredinia of *Austropuccinia psidii* on leaves of *Psidium guajava* and *Syzygium jambos* in Brazil (Silva et al. 2023) and uredinia of *Hemileia vastatrix* on leaves of *Coffea arabica* from Africa (Pereira et al. 2024). This is the first report of *C. guizhouense* from aphids on *Telosma cordata* (*Apocynaceae*). The scale insect *Kuwanaspis howardi* is commonly distributed and harmful to bamboo. In our previous study, two *Podonectria* species and a *Microcera* species associated with native bamboo plants were discovered (Xu et al. 2021a). *Cladosporium kuwanaspidis* is the second entomopathogenic species found on bamboo (*Pleioblastus amarus*) in our investigations. As entomopathogenic *C. cladosporioides* is a potential candidate for biocontrol against whiteflies and aphids (Islam et al. 2019; Nicoletti et al. 2024), and as endophytic *Cladosporium* sp. has demonstrated insecticidal activity of α-glucosidase inhibitors against *Spodoptera litura* in vitro (Singh et al. 2015), more *Cladosporium* species need to be investigated as potential agents of biocontrol.

The genus *Moelleriella* (*Ascomycota*, *Hypocreales*, *Clavicipitaceae*) was established to accommodate *M. sulphurea* (Bresadola 1896) and is characterized by globose to pyriform perithecia immersed in a brightly colored stroma with openings protruding from the stroma on scale insects (*Coccidae*, *Homoptera*) or whiteflies (*Aleyrodidae*, *Homoptera*), as well as multi-septate ascospores that disarticulate at the septa inside the ascus. In contrast, species of *Hypocrella* and the subsequently proposed genus *Samuelsia*, having *Aschersonia*-like anamorphs, have ascospores that remain whole (Petch 1921; Mains 1959; Kirk et al. 2001; Chaverri et al. 2008). The genus currently has about 52 records (https://www.speciesfungorum.org/Names/Names.asp, accessed on 4 November 2024), and species have been reported in Belize, Bolivia, Costa Rica, Côte d’Ivoire, China, Ecuador, Ghana, Guatemala, Guiana, Honduras, Jamaica, Mexico, Panama, the Philippines, Thailand, Trinidad, Venezuela, and Vietnam, frequently from Thailand, indicating it is more common in tropical regions (Liu et al. 2006; Chaverri et al. 2008; Qiu et al. 2009; Mongkolsamrit et al. 2011b, 2015; Li et al. 2016; Tibpromma et al. 2017; Chen et al. 2020; Yuan et al. 2020; Khonsanit et al. 2021). *Moelleriella libera* has been widely used due to its parasitism of large populations of whiteflies and scale insects in the wild (Zhang et al. 2018; Ingle et al. 2019; Qasim et al. 2020). In China, *M. ochracea* has previously been recorded from Fujian Province on homopteran cadavers (Qiu et al. 2009). In recent years, several species have been reported from the subtropical regions of Yunnan and Fujian provinces, viz. *M. gracilispora* on whitefly nymphs (Hemiptera) (Yuan et al. 2020), *M. sinensis* infecting whitefly nymphs (Hemiptera) (Chen et al. 2020), *M. puerensis* on whiteflies (Wang et al. 2022c), *M. simaoensis* on whiteflies (Yang et al. 2023a), and *M. jinuoana* and *M. longzhuensis* on scale insects and whiteflies (Wang et al. 2024). Based on our observation, four *Moelleriella* species were newly recorded on scale insects inhabiting *Boehmeria spicata*, *Citrus* sp., and *Eucalyptus* sp. in Sichuan Province. *Moelleriella sinensis*, *M. cinnamomum*, and *M. citrus* were accommodated in the Effuse clade (Chaverri et al. 2008; Wang et al. 2024), which is characterized by effuse to thin, pulvinate stromata with loose hyphal tissue, mostly having hypothalli. The new species *M. boehmeriae* was nested in the Globose clade (Chaverri et al. 2008), characterized by globose, darker stromata with compact tissue.

*Podonectriaceae* was proposed as a family in the *Pleosporales* to accommodate the genus *Podonectria*, which was confirmed by phylogenetic analyses (Dao et al. 2016; Yang et al. 2019b). *Podonectria* was traditionally recorded with a *Tetracrium*-like conidial stage (Petch 1921; Rossman 1978). Previous studies confirmed the link between the sexual morphs in *Podonectria* and the asexual morphs in *Tetracrium* with identical molecular sequences (Dao et al. 2015, 2016; Xu et al. 2021a). In addition, an asexual genus, *Tetranacrium*, typified by *T. gramineum* (Hudson and Sutton 1964), was recorded as an associated anamorph of *Podonectria gahnia* on scale insects. However, although the shape and development of conidia in the associated *Tetranacrium* are correlated with *Tetracrium*, the conidiomata in *Tetranacrium* are pycnidia, whereas in *Tetracrium* they are sporodochia. The relationship between *Tetranacrium* and *Podonectria* requires further phylogenetic and taxonomic studies with more samples. In this study, our collections had hyphomycetous anamorphs, which were consistent with *Podonectria*, identified based on the phylogenetic results of combined ITS, LSU, SSU, *tef*1-α, and *rpb*2 data. Most species of *Podonectria* are associated with armored scale insects on *Citrus* spp. *Podonectria multiarmata* is the third entomopathogenic species associated with *Kuwanaspis howardi* on bamboo (*Pleioblastus amarus*) in China.

## ﻿Conclusion

Seven entomopathogenic fungi from Sichuan Province, China, are described, including six new species and one newly recorded species. These species are morphologically simple hyphomycetes or coelomycetes, with detailed identification based on morphological characteristics and phylogenetic analyses. These fungi warrant attention, particularly those associated with major agricultural and forestry pests. We predict that southwestern China harbors a rich diversity of entomopathogenic fungi, with many species yet to be discovered and evaluated. These fungi represent a crucial resource for future drug development and hold significant potential for pest management in agriculture and forestry.

## Supplementary Material

XML Treatment for Cladosporium kuwanaspidis

XML Treatment for Cladosporium guizhouense

XML Treatment for Moelleriella eucalypti

XML Treatment for Moelleriella boehmeriae

XML Treatment for Moelleriella cinnamomum

XML Treatment for Moelleriella citrus

XML Treatment for Podonectria multiarmata

## ﻿Additional information

### Use of AI

No use of AI was reported.

### Author ORCIDs

Chunlin Yang https://orcid.org/0000-0002-3854-8835

Xiulan Xu https://orcid.org/0000-0002-6832-5421

### Data availability

All sequence data generated for this study are available in GenBank (https://www.ncbi.nlm.nih.gov/genbank/).
